# Proceedings of the Twentieth International Society of Sports Nutrition (ISSN) Conference and Expo

**DOI:** 10.1080/15502783.2023.2235311

**Published:** 2023-07-27

**Authors:** Chad M. Kerksick, Trisha VanDusseldorp, Douglas Kalman, Jose Antonio

**Affiliations:** a Lindenwood University, Exercise and Performance Nutrition Laboratory, Kinesiology Department, College of Science, Technology, and Health; b Bonafide Health, LLC p/b JDS Therapeutics, Harrison, New York, NY, USA; c Nova Southeastern University, Department of Nutrition, Davie, FL, USA; d Nova Southeastern University, Department of Health and Human Performance, Davie, FL, USA

**Body Composition Differences by Position in Professional Baseball Players**

Luis Rios-Jiminez^a,b^, Jonothan Freeston^b^, Lucas VanDyke^b^, Gabriel J. Sanders^c^,Tobin Silver^a^, Jose Antonio^a^, Corey A. Peacock^a^*

^a^Department of Health and Human Performance, Nova Southeastern University, Davie FL USA; ^b^Cleveland Guardians, Cleveland OH USA; ^c^Department of Kinesiology, Northern Kentucky University, Highland Heights KY USA

*Corresponding author: cpeacock@nova.edu

**Background**: Previous research demonstrates body composition comparisons relative to their dominant arm vs non-dominant arm in collegiate baseball players. Further research demonstrates body composition comparisons relative to their position in collegiate baseball players. Though there is a vast amount of literature on nonprofessional baseball players, there is little research that analyzes body composition in professional baseball players. Therefore, the purpose of this study is to compare body composition variables by position in professional baseball players. We hypothesize that differences will exist in body mass and composition between positions.

**Methods**: 178 (n = 60 Infield, n = 23 Outfield, n = 95 Pitcher) professional baseball players (22.3 ± 3.1 yrs.; 184.8 ± 6.7 cm) were used for the current study. The assessment was collected in a noninvasive bioelectric impedance analyzer machine (InBody770), in which the athlete stepped on the scale in the morning, before eating for at least 2 hours prior to the assessment and was well hydrated. Hydration levels were measured through an osmolality meter (Atago PAL-mOsm) with a score of no higher than 600 mOsmol/kgH2O. One-Way ANOVA was utilized to analyze the main effects of the group. The Bonferroni Post Hoc analysis was utilized to compare positions, and significance was set at P ≤ 0.05.

**Results**: There is a significant main effect of the group for body fat percentage (BF) (P = .007), body weight (BW) (P < .001), fat-free mass (FFM) (P < .001), and fat mass (FM) (P = .004) in professional baseball players. Post hoc analysis revealed further comparisons between Infield vs Outfield (Table 1.), Infield vs Pitcher (Table 2), and Outfield vs Pitcher (Table 3).

**Table 1.** Infield vs. outfield.

	Infield	Outfield	P-VALUE
BF (%)	14.6 ± 4.8	11.6 ± 4.2	*P = 0.033
BW (kg)	86.7 ± 9.7	88.9 ± 10.4	P = 1.000
FFM (kg)	73.9 ± 7.5	78.3 ± 7.0	*P = 0.037

M± SD, *Significance set at P ≤ 0.05

**Table 2.** Infield vs. pitcher.

	Infield	Pitcher	P-VALUE
BF (%)	14.6 ± 4.8	15.1 ± 4.9	P = 1.000
BW (kg)	86.7 ± 9.7	94.9 ± 9.6	*P < .001
FFM (kg)	73.9 ± 7.5	80.3 ± 7.0	*P < .001

M± SD, *Significance set at P ≤ 0.05

**Table 3.** Outfield vs. pitcher.

	Outfield	Pitcher	P-VALUE
BF (%)	11.6 ± 4.2	15.1 ± 4.9	*P = 0.005
BW (kg)	88.9 ± 10.4	94.9 ± 9.6	*P – 0.028
FFM (kg)	78.3 ± 7.0	80.3 ± 7.0	P = 0.701

M± SD, *Significance set at P ≤ 0.05

**Conclusion**: Professional baseball players’ body composition varies significantly based on the positions and the different demands of the sport. Based on the data, the infielders have significantly less BF than the outfielders, while the outfielders have significantly more FFM. The data also suggest that Pitchers have significantly higher BW and FFM when compared to Outfielders. No other significant differences were determined in the current study between positions. Future research aims to analyze the body composition data based on the throwing arm.

**Body Composition Differences by Position in Football Players Preparing for the NFL Combine**

Angie Dusak^a^, Arzoo Hassanzada^a^, Jose Antonio^a^, Corey A. Peacock^a^*

^a^Department of Health and Human Performance, Fight Science Lab, Nova Southeastern University, Davie FL USA

*Corresponding author: cpeacock@nova.edu

**Background**: Body composition measurements are instrumental in aiding sports performance coaches. Improvements in body composition may contribute to the overall increased performance, including speed, endurance, and reaction time. Football positions have been shown to be associated with body composition specific to the demands of the position. Previous research has shown differences in both total and regional body composition measures between player positions in NFL players. The purpose of this study is to compare body composition measures by player position in players preparing for the NFL Combine. We hypothesize that differences will exist in body composition between players of different position groups.

**Methods**: A total of 28 (n = 16 Skill, n = 10 Big Skill, n = 2 Big) NFL combined players (21.8 ± 1.3 yrs.; 185.6 ± 6.1 cm) were measured for this study (Skill = running back, wide receiver, defensive back; Big Skill = quarterback, fullback, tight end, linebacker; Big = offensive linemen, defensive linemen). Body composition was measured using a noninvasive InBody Machine (InBody270). Athletes stepped onto the scale and held onto the handles, while the InBody used bioelectrical impedance technology to measure body composition. Means and standard deviations were calculated for descriptive data. Univariate ANOVA was used to analyze the main effects of the group. The Bonferroni Post Hoc analysis was utilized to compare player positions, and the significance was set at P ≤ 0.05. All statistics were analyzed using Statistical Analysis Software (SPSS, Version 22.0, IBM INC., Armonk, NY).

**Results**: All measures of body composition (height, weight, total body water, dry lean mass, body fat mass, lean body mass, skeletal muscle mass, LBM left arm, LBM right arm, LBM trunk, LBM left leg, and LBM right leg) were significantly (p < 0.05) different by player position between Skill vs. Big Skill players and Big Skill players. There was no difference in measures of body composition between Big vs. Big Skill players.

**Conclusions**: There are clear differences in body composition among football players preparing for the NFL Combine.

**The Effects of a Sports Nutrition Education Intervention on Body Composition and Resting Metabolic Rate in NCAA Division I Men’s Soccer Players**

K. Michelle Singleton^a^*, Jamie McAllister-Deitrick^a^, Keirstin Roose^a^, Chad Kerksick^b^, Kelly Johnson^a^

^a^Department of Kinesiology, Coastal Carolina University; ^b^Exercise and Performance Nutrition Laboratory, Lindenwood University

*Corresponding author: ksingleto@coastal.edu

**Background**: The overall purpose of this study was to investigate the effects of an 8-week sports nutrition education intervention on body composition and resting metabolic rate (RMR) within Division I (DI) men’s soccer players. Athletes typically have a higher energy requirement compared to the general population which can make it difficult for them to consume enough energy. Appropriate energy intake supports optimal body function, fuels physical work, facilitates repair and recovery, and is a key consideration in manipulating body composition. Previous studies suggest that collegiate athletes have poor sports nutrition knowledge and, as such, diets followed by collegiate athletes are insufficient in total energy, macronutrients, micronutrients, and fluids resulting in problems maintaining energy balance.

**Method**: National Collegiate Athletic Association Division I men’s soccer players (n = 16) participated in the study during in-season training and matches. The intervention consisted of four in-person, biweekly (8 weeks total) sports nutrition education sessions focusing on macronutrients, micronutrients, hydration, and supplements. Body composition and RMR were measured pre- and post-intervention. RMR was predicted using the Nelson equation. Body composition was measured using air displacement plethysmography (Bod Pod), and RMR was measured using indirect calorimetry.

**Results**: Paired sample t-tests were conducted to compare differences in pre- and post-intervention. Following the intervention and sports season, there was a significant increase (*p*= 0.001) in overall body mass (pre, 71.9 ± 7.9; post, 73.0 ± 9.2) and body fat percentage (*p* =  0.003) (pre, 8.4 ± 2.5; post, 10.1 ± 2.6), whereas RMR decreased (*p* =  0.049) following the intervention and season (pre, 2,031 ± 224; post, 1,923 ± 231). Additionally, the Bod Pod predicted RMR and measured RMR were compared. Measured RMR (in kcals/day) was significantly higher (*p* < 0.001) compared to predicted RMR at pre (estimated: 1,722 ± 189 vs. measured: 2,031 ± 224) and post (estimated: 1,241 ± 212 vs. measured: 1,923 ± 231).

**Conclusions**: In this pre- and post-intervention study evaluating body composition and RMR among male DI soccer athletes, the increase in overall body mass and body fat percentage along with the decrease in RMR indicates poor nutritional habits throughout the sports season. Additionally, the significant difference between predicted and measured RMR reinforces the need to measure RMR when possible. Further research on sports nutrition education interventions is recommended, specifically assessing sports nutrition knowledge and dietary behavior among this population is warranted.

**No Jitters, but No Energy**

Kristiina Kinnunen^a^, Cassandra Evans^a,b^, Jason Curtis PhD^c^, Flavia F. Pereira^b,c^, Kendall Andries^a^, Rachel DeBlasio^a^, Viraaj Miriyala^a^, Jose Rojas^b^, Maria Berrocales^a^, Juan Carlos Santana^d^, Jose Antonio PhD^a,d^*

^a^Exercise and Sport Science, NSU Florida, Davie, FL 33328, USA; ^b^Health Sciences, Rocky Mountain University of Health Professions, Provo, UT 84606, USA; ^c^Exercise and Sports Science, Keiser University Flagship, West Palm Beach, FL 33409, USA; ^d^Institute of Human Performance, Boca Raton, FL 33432, USA

*Corresponding author: jose.antonio@nova.edu

**Background**: The purpose of this investigation was to determine the effects of an energy drink (*Jocko GO*) on measures of mood, sustained attention/reaction time, and hand steadiness.

**Methods**: A total of 22 active men (n = 6) and women (n = 16) (mean ± SD: age 22 ± 5 yr; height 169 ± 9 cm; body mass 68 ± 9 kg; lean body mass 52 ± 10 kg; fat mass 15 ± 7 kg; percent body fat 22 ± 9%; total body water 38 ± 7 liters) participated in this randomized, crossover, counterbalanced trial. Research participants arrived at the Institute of Human Performance (IHP) on two different occasions separated by one week. The following assessments were conducted: psychomotor vigilance test, Profile of Mood States (POMS), and hand steadiness test (Hole Type Steadiness Tester, Lafayette Instrument) to test for the “jitters.” Each subject consumed either one can (355 ml) of the energy drink or consumed nothing (i.e. control condition). Each assessment was made 30 minutes after consuming the energy drink, whereas, in the control condition, assessments were made 30 minutes after arrival to IHP.

**Results**: There were no significant differences between the control and treatment for any of the assessments (Table 1). All data are expressed as the mean ± SD.

**Conclusions**: This particular energy drink does not cause the ”jitters.” However, it has no effect on sustained attention/reaction time or mood (i.e., vigor, fatigue). Future research should examine additional time points as well as different energy drink doses.

**Weigh-In versus Fight-Weight in Professional Mixed Martial Artists Competing in the Heavyweight Division**

Corey A. Peacock^a^*, Gabriel J. Sanders^b^, Anthony Ricci^a^, Charles Stull^c^, Duncan French^c^, Cassandra Evans^a^, Jose Antonio^a^

^a^Department of Health and Human Performance, Nova Southeastern University, Davie FL USA; ^b^Department of Kinesiology, Northern Kentucky University, Highland Heights KY USA; ^c^UFC Performance Institute, Las Vegas NV USA

*Corresponding author: cpeacock@nova.edu

**Background**: Previous research has demonstrated that professional mixed martial artists (MMA) employ a variety of weight manipulation strategies in order to compete at a given weight-class. Although there is much literature demonstrating weight manipulation methods, minimal research exists analyzing how much weight professional MMA gains between the official weigh-in and competition. Therefore, the purpose of the current study is to compare the official weigh-in and fight-weight , specifically, in professional MMA competing in the heavyweight division.

**Methods**: Thirty-eight (38) professional heavyweight (120.2 kg limit) MMA (32.5 ± 4.3 yrs.; 190.1 ± 4.9 cm) fighters competing for the Ultimate Fighting Championship (UFC) between 2020 and 2022 were used for the study. The athletes reported to the arena for an official weigh-in (24-36 hours prior to competition). An official weight was obtained utilizing the commission managed beam scale. The following day, athletes return to the arena for competition, and weights are obtained using a commission calibrated digital scale. Paired t-tests were utilized, and the significance was set at P ≤ 0.05.

**Results**: There is a non-significant (P = 0.462) difference between weigh-in and fight-weight in professional heavyweight MMA (Table 1).

**Conclusion**: Unlike other weight-divisions, it appears that professional heavyweight MMA competing for the UFC does not increase body weight following official weigh-ins. Further data is being analyzed to better understand rehydration protocols in professional MMA.

**Weight Loss in Female Professional Mixed Martial Arts Fighters 24 Hours Prior to Official Weigh-In**

Gabriel J. Sanders^a^*, Anthony Ricci^b^, Charles Stull^c^, Duncan French^c^, Jose Antonio^b^, Cassandra Evans^b^, Corey A. Peacock^b^

^a^Department of Kinesiology, Northern Kentucky University, Highland Heights KY USA; ^b^Department of Health and Human Performance, Nova Southeastern University, Davie FL USA; ^c^UFC Performance Institute, Las Vegas NV USA

*Corresponding author: sandersg1@nku.edu

**Background**: Previous research has shown that professional mixed martial arts fighters (MMA) utilize a variety of strategies to manage and lose weight prior to officially weighing in for competition. Although there is much literature demonstrating weight loss and methods, minimal research exists analyzing how much weight a professional female MMA fighterloses 24 hours prior to the official weigh-in. Therefore, the purpose of the current study is to compare professional female MMA weight 24 hours prior versus official weigh-in.

**Methods**: One hundred twenty-eight (128) professional female MMA (31.4 ± 4.4 yrs.; 164.9 ± 5.4 cm) fighters competing for the Ultimate Fighting Championship (UFC) between 2020 and 2022 were used for the study. Fighters from all women’s UFC weight divisions were utilized [strawweight (52.2 kg): flyweight (56.7 kg); bantamweight (61.2 kg); featherweight (65.8 kg)]. The fighters reported to the assigned workout rooms 24 hours prior to the official weigh-ins. Weights were obtained using a commission calibrated digital scale. The following day, an official weigh-in took place where weight was obtained utilizing the official commission managed beam scale. A pair t-test was utilized, and the significance was set at P ≤ 0.05.

**Results**: There is a significant (P < .001) weight difference between 24 hours pre and the official weigh-in (Table 1).

**Conclusions**: Female MMA fighters competing for the UFC decrease their body weight significantly 24 hours prior to official weigh-ins. Based on these data, it appears the athletes averaged a weight loss of nearly 4.6% prior to weigh-ins. Further data is being analyzed to better explain weight loss strategies in professional female MMA.

**The Effects of a Nutrition Education Program on Athletes’ Nutritional Knowledge, Eating Habits, and Performance**

Nagham Sannan^a^*, Hanine Bou Antoun^b^ Nour El Helou^a^

^a^Laboratory of Human Nutrition, Nutrition Department, Faculty of Pharmacy, Saint Joseph University of Beirut, Beirut, Lebanon; ^b^College of Humanities and Sciences, Ajman University, Ajman,UAE

Corresponding author: nagham.sannan@net.usj.edu.lb

**Background**: This study aims to assess the impact of a nutrition education program on Lebanese athletes’ nutritional knowledge, eating habits, and performance outcomes.

**Methods**: A sample of 200 athletes were divided into two groups: an intervention group (IG; N = 151, age: 30.1 ± 5.7 years; height: 179.4 ± 8.2 cm) and a control group (CG; N = 47, age: 31.9 ± 5.8 years; height 174.4 ± 7.8 cm). The intervention group followed a 4-month nutritional counseling program, which included three nutritional education lectures and individual consultations with athletes. The same nutrition intervention was repeated 10 months later for the IG. The athletes’ eating habits, nutrition knowledge, body composition, and performance were evaluated at the beginning and at the end of the protocol through a food frequency questionnaire, a knowledge assessment questionnaire, and dietary recalls. Several approaches were used to evaluate the performance of athletes, such as the beep test, maximum aerobic speed (VMA), and 1 repetition maximum (RM).

**Results**: The nutritional knowledge scores (NKS) of the IG increased by 31.1%, indicating a significant improvement in their understanding of proper nutrition (p < 0.001). Both groups reduced their salt intake, but the IG demonstrated a more significant reduction compared to the CG (IG reduced salt intake by 24.6%; p < 0.001 versus CG which reduced the salt intake by 13%). The IG showed a 51.2% percent increase in demand for nutritional counseling (p < 0.001). The proportion of athletes who read the nutrition facts labels increased by 34.9% (p < 0.001). The IG also demonstrated a 64.1% (p < 0.001) increase in the percentage of athletes who obtained their nutritional information from registered dietitians. The IG showed a significant decrease in the percentage of body fat, with a reduction of 5.4% (p = 0.009). The CG revealed an increase in the percentage of body fat by 14.4%. Concerning sports performance, the IG displayed an increase of 2.6% in the maximal aerobic speed VMA (km/hr.) (p < 0.001) and a 2.6% increase in the maximal oxygen uptake VO_2_ max (ml/kg/min), (p < 0.001).

**Conclusion**: The results of the study indicate that the nutritional intervention had a positive impact on the athletes’ nutritional knowledge, eating habits, body composition, and performance. Compared to previous research, our study adopts an interactive and intensive educational intervention, involves a control group, and analyzes athletes across multiple factors. This approach makes our study distinctive and contributes to the improvement of athletes’ health and performance.

**Comparative Differences of Amino Acid Appearance, With and Without Probiotic Ingestion**

Christine Florez^a^, Dylon Miller^a^, Mandy Parra^a^, Jaci Davis^a^, Jessica Prather^a^, Abby Harrison^a^, Amie Vargas^a^, Audrey Ross^a^, Bella Soto^a^, Ralf Jäger^b^, Martin Purpura^b^, Grant Tinsley^c^ & Lem Taylor^a^*

^a^University of Mary Hardin-Baylor, Belton, TX; ^b^Increnovo LLC, Whitefish Bay, WI^c^Texas Tech University, Lubbock, TX

*Corresponding author: ltaylor@umhb.edu

**Background**: Ingestion of certain probiotic strains has been shown to alter the absorption rates of amino acids following acute protein ingestion. Protein uptake has been shown to be paramount to the growth and maintenance of optimal body composition and overall health. The purpose of this study was to assess if supplementation of the probiotic *Bacillus subtilis* would alter the absorption of branched-chain amino acids (BCAAs), essential amino acids (EAAs), and total amino acids following the ingestion of plant-based proteins.

**Materials and Methods**: Sixteen male (n = 16; age: 23.1 ± 3.2 y; height: 180.3 ± 7.7 cm; weight 86.6 ± 13.8 kg; body fat 17.8 ± 6.3%) participants co-ingested 26.7 g pea protein yielding 20 g of protein with either *Bacillus subtilis* or a placebo for two weeks in a randomized, double-blind, crossover design study separated by a 4-week washout period. Blood samples were taken during the first laboratory visit, prior to the two-week supplementation period. During the second visit, samples were taken at a baseline of 30, 60, 120, and 180-minutes post-ingestion of a 20-gram plant-based protein powder bolus. Plasma amino acid levels were analyzed by using liquid chromatography and tandem-mass spectrometry (LC/MS/MS). The maximum observed concentration (Cmax) and area under the concentration vs. time curve (AUC) were calculated. The Cmax and AUC for total amino acids, EAA, and BCAA were compared between the active treatment and placebo groups using Wilcoxon signed-rank tests, and Wilcoxon *r* effect sizes were calculated. Safety of supplementation was evaluated through a standard blood panel and reported side effects.

**Results**: No differences between conditions were observed for the Cmax of total amino acids ([median ± IQR] active: 3601 ± 353, placebo: 3539 ± 561 umol/L; p = 0.94, r = 0.03), EAA (active: 1462 ± 156 umol/L, placebo: 1482 ± 394 umol/L; p = 0.71, r = 0.04), and BCAA (active: 652 ± 173 umol/L, placebo: 656 ± 161 umol/L; p = 0.49, r = 0.18). Similarly, no differences were observed for the AUC of total amino acids (p = 0.71, r = 0.1), EAA (p = 0.74, r = 0.09), and BCAA (p = 0.74, r = 0.09). No relevant effects of treatment on blood panel safety markers nor serious adverse effects were observed.

**Conclusions**: Ingestion of *Bacillus subtilis* did not alter amino acid absorption following an acute ingestion of dietary protein. Consumption of this probiotic strain appears to be safe.

**Effects of Astaxanthin on Inflammatory and Oxidative Stress Responses to Fire Suppression Activities**

Drew E. Gonzalez^a^*, Courtney C. Dillard^b^, Megan Leonard^c^, Broderick L. Dickerson^a^, Choongsung Yoo^a^, Joungbo Ko^a^, Dante Xing^a^, Victoria Martinez^a^, Jacob Kendra^a^, Sarah E. Johnson^a^, Landry Estes^a^, Ryan J. Sowinski^a^, Chris J. Rasmussen^a^, Steven E. Martin^c^, Matthew J. McAllister^b^, Richard B. Kreider^a^

^a^Exercise and Sport Nutrition Laboratory, Department of Kinesiology and Sport Management, Texas A&M University, College Station, TX; ^b^Metabolic and Applied Physiology Laboratory, Department of Health and Human Performance, Texas State University, San Marcos, TX; ^c^Sydney and JL Huffines Institute for Sports Medicine and Human Performance, Department of Health and Kinesiology, Texas A&M University, College Station, TX

Corresponding author: dg18@tamu.edu

**Background**: Firefighters are at risk for cardiovascular disease (CVD) or mortality due to specific stressors encountered on duty. Natural antioxidant/anti-inflammatories, such as astaxanthin (AX), have been purported to reduce oxidative stress and inflammation, which contribute to CVD risk; however, minimal work has been done among firefighters. The purpose of this study was to examine the effects of 4-weeks of AX supplementation on inflammatory and oxidative stress responses to a simulated fire grounds test (FGT).

**Methods**: In a randomized, double-blinded, placebo-controlled, crossover fashion, 15 career, male firefighters (age: 34.5 ± 7.4 yr, body mass: 95.6 ± 12.0 kg, height: 177.7.8 ± 7.0 cm, VO_2max_: 41.0 ± 5.9 ml/kg/min) ingested 12 mg/d of AX (AstaReal®, Burlington, NJ, USA) or placebo (PLA) for four weeks separated by a 7-14-day washout period while participating in a standardized resistance and conditioning program. Following each supplementation period, participants completed an FGT consisting of nine firefighter-specific tasks. Participants performed the FGT while dressed in full bunker gear and were “on air.” Saliva samples were collected 30-min prior to the FGT (Pre-30), 5-min before the FGT (Pre-5), following the FGT (Post-FGT), and after 30-min of recovery (30-Rec) and assayed for interleukin-1β (IL-1β), cortisol (CORT), and uric acid (UA). In addition, air utilization during the FGT was assessed. Data were analyzed using a general linear model multivariate analysis with repeated measures and partial eta squared ($\eta_{p}^{2}$) values to assess effect-size. Mean changes from the baseline with 95% confidence intervals (mean [LL, UL]) were used to assess clinical significance.

**Figure 1.**
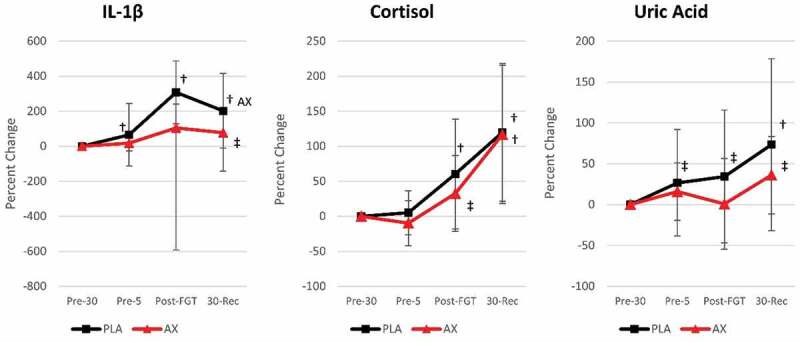
Fire grounds test salivary responses.

**Results**: The GLM analysis revealed no significant overall or univariate treatment × time interaction effects for salivary IL-1β, CORT, UA, or air utilization during the FGT. However, analysis of mean changes from the baseline revealed that AX delayed and/or lessened the increase in IL-1β, CORT, and UA response to the FGT. For example, salivary IL-1β significantly increased above the baseline at Pre-5, Post-FGT, and 30-Rec with PLA treatment, whereas values were only increased above baseline with AX at 30-Rec and tended to be lower than PLA 30-Rec values (−124 [−253, 4.9] %, *p* =  0.059, $\eta_{p}^{2}$ = 0.122, strong effect). Similarly, UA levels significantly increased above baseline at each datapoint with PLA, but only tended to be higher than baseline after 30-Rec with AX (36.1 [−7.1, 79.2] %, *p* = 0.098, $\eta_{p}^{2}$ = 0.05, weak to moderate effect) (Figure 1).

**Conclusions**: Results provide some evidence that AX may lessen the inflammatory responses to fire suppressive training demands. However, additional research is warranted. Firefighters may benefit by increasing the availability of astaxanthin in their diet as a means of lessening inflammation and/or oxidative stress associated with performing firefighting activities. Additional research is needed to determine if this has a long-term impact on cardiometabolic health in firefighters.

**Acknowledgments**: We would like to thank the local Bryan Fire Department for their participation in this study. This study was funded by AstaReal® (Burlington, NJ, USA).

**An acute, single-dose treatment of Caffeine, TeaCrine, and Dynamine improves neurophysiological and performance measures of e-gamers**

Casandra Evans^a,b^, Jose Antonio^b^, Amani Khan^c^, Samir Sakaria^c^, Alexandra Vanderkley^c^, Maria Berracoles^b^, Jose Rojas^b^, Juan Carlos Santana^d^, Jason Curtis^e^, Joseph Petruzelli^b^, Tony Ricci,^b^ and Jaime L. Tartar^c^

^a^Nutrition/Exercise and Sports Sciences, Rocky Mountain University of Health Professions, Ft. Lauderdale, USA; ^b^Nova Southeastern University, Exercise and Sport Science, 3301 College Ave, Ft Lauderdale, FL, 33,314, USA; ^c^Nova Southeastern University, College of Psychology, 3301 College Ave, Ft Lauderdale, FL, 33,314, USA; ^d^Institute of Human Performance, Boca Raton, USA; ^e^Exercise and Sports Science, Keiser University, West Palm Beach, USA

Corresponding author: Jose.Antonio@nova.edu

**Background**: The primary aim of this study was to evaluate the effects of a combined Caffeine + TeaCrine® + Dynamine™ on measures of neurophysiological activity, as well as performance, during first-person shooter games in e-gamers.

**Methods**: Using a randomized double-blinded, crossover design, we assessed the effects of an acute, single-dose treatment of Caffeine (200 mg) vs. Caffeine (200 mg) + TeaCrine (10 mg) + Dynamine (50 mg) (CTD) vs. Placebo (maltodextrin). Each participant was tested under all three conditions 1 week apart. Baseline and post-dose measures were tested 1 hour apart. Participants [n = 49 male (24.4 ± , 4.5 yr)] were amateur e-gamers who played a first-person video game at least 10 hrs/week. Gaming performance was assessed through a series of first-person shooter training games through AIMLAB (State Space Labs, Inc.) These included Reflex Shot (RS) Standard, Speed, and Precision. Neurophysiological activity was captured when participants played three games through a single-channel EEG (Enchanted Wave, LLC) with the electrode positioned at the prefrontal-lobe location (Fp1).

**Results**: In the RS Standard game, analyses of targets showed that while all conditions increased pre- and post-dose, the CTD condition shot significantly more targets (i.e. had better performance) relative to both caffeine (p = 0.01) and placebo (p = 0.03) post-dose. In the RS Speed game, there was a significantly greater number of targets shot in the post-dose testing compared to the pre-dose testing in Caffeine (p = 0.01) and Placebo (p = 0.02). There were significantly more targets hit post-dose compared to pre-dose in the Caffeine (p = 0.03) and CTD (p = 0.004). Only the Placebo condition had a significant increase in the kills per second post-dose compared to pre-dose (p = 0.04), and the CTD group had significantly fewer total kills post-dose compared to pre-dose (p = 0.02). In the RS Precision game, all conditions significantly improved on the number of targets in the post-dose testing compared to the pre-dose testing (all p-values < 0.05). While the caffeine condition took significantly more shots post-dose compared to pre-dose, only the CTD condition significantly improved the number of kills per second post-dose (p = 0.01). EEG data collected concomitantly with game playing showed that the CTD condition resulted in significantly lower alpha power compared to the Placebo condition (p = 0.02). The CTD group also showed increased theta activity post-dose during game playing compared to both the Placebo (p < 0.001) and Caffeine (p < 0.001) conditions.

**Conclusions**: CTD appears to improve overall shooting gaming performance and neurophysiological measures of cognitive activity compared to Caffeine and Placebo. Collectively, these findings suggest that CTD assists with speed-accuracy tradeoffs where caffeine-only can lead to erratic play; thus, CTD may be particularly beneficial for shooting precision. The EEG data support this notion since the CTD exhibited lower alpha power, suggesting increased cognitive flexibility and arousal, and higher theta power, suggesting greater cognitive control and decision making under pressure.

**Acknowledgments**. This research was funded by Compound Solutions (Carlsbad, CA). The funder played no role in data acquisition, data management, nor data interpretation.

**Bodybuilding Coaching Strategies Meet Evidence-Based Recommendations: A Qualitative Approach**

Alexa Rukstela^a^, Kworweinski Lafontant^b^, Eric Helms^b,c^, Guillermo Escalante^d^, Kara Phillips^a,e^, Bill I. Campbell^a^*

^a^Exercise Science Program, University of South Florida, Tampa, FL, 33,620; ^a^Sports Performance Research Institute New Zealand, Auckland University of Technology, Auckland 1010, New Zealand; ^c^Florida Atlantic University, Department of Exercise Science and Health Promotion, Muscle Physiology Laboratory, Boca Raton, FL, United States; ^d^Department of Kinesiology, California State University, San Bernardino, San Bernardino, CA, 92,407; ^e^Department of Kinesiology, Nutrition, and Dietetics, University of Northern Colorado, Greeley, CO, 80,639

*Corresponding author: bcampbell@usf.edu

**Background**: Bodybuilding is a sport where athletes are judged on esthetic qualities of their physique such as muscle size, low body fat, and overall symmetry. To achieve this look, bodybuilders typically train for years to increase muscle mass, then enter a preparation/dieting phase, with the aim of reducing body fat to extremely low levels while maintaining high levels of muscularity. Previous bodybuilding survey studies regarding common practice within the sport have been investigated from the perspective of the athletes themselves. To the authors’ knowledge, little attention has been given to the bodybuilding coaches who often orchestrate these competition preparations. The present study sought to gain an understanding of common decisions and rationales employed by bodybuilding coaches.

**Methods**: Focusing on coaches of the more muscular divisions in the National Physique Committee/IFBB Professional League federations (men’s classic physique, men’s bodybuilding, women’s physique, women’s bodybuilding) for both natural and enhanced athletes, coaches were recruited via word-of-mouth and social media, and 33 responded to an anonymous online survey. Participant coaches were allowed to skip any question they did not want to answer or feel qualified to answer based on their background and experiences.

**Results**: Survey responses indicated that participant coaches recommended three to seven meals per day and no less than 2 g/kg/day of protein regardless of sex, division, or performance enhancing drug (PED) usage. During contest preparation, participant coaches alter a natural competitor’s protein intake by – 25% to +10% and an enhanced competitor’s protein intake by 0% to +25%. Regarding cardiovascular exercise protocols, approximately two-thirds of participant coaches recommend fasted cardiovascular exercise, with the common rationale of combining the exercise with thermogenic supplements while considering the athlete’s preference. Low and moderate intensity steady states were the most commonly recommended types of cardiovascular exercise among participant coaches; high-intensity interval training was the least popular. Creatine was ranked in the top-2 supplements for all surveyed categories. Regarding PEDs, testosterone, growth hormone, and methenolone were consistently ranked in the top-5 recommended PEDs by participant coaches.

**Conclusions**: No two bodybuilding coaches answered exactly the same, which speaks to the nuance of methodology in bodybuilding coaching. While the results of this study provide insight into common themes in the decisions made by bodybuilding coaches, it also highlights areas in which more research is needed to empirically support those decisions, especially regarding supplementation, protein intake, and PEDs.

**Acknowledgments**: The authors declare no conflicts of interests. There was no funding support for this study.

**Hormonal, Morphological, and Psychological Changes During Contest Preparation for a First-Time Natural Bikini Competitor**

Alexa Rukstela, Kworweinski Lafontant, Bill I. Campbell*

Exercise Science Program, College of Education, University of South Florida, Tampa, FL, 33,620

*Corresponding author: bcampbell@usf.edu

**Background**: The purpose of this case report was to document the physiological and psychological changes that occurred throughout a 6-month preparation and 1-month post-competition period for a true novice natural bikini competitor. Case reports provide insight into the strategies employed by bodybuilders, which can be beneficial to coaches and athletes in their decision-making processes. Despite this value, there is a paucity of data on female true novice competitors in any division as well as psychological consequences of a preparation phase.

**Methods**: A private coach developed the athlete’s nutritional and training protocols. The diet throughout preparation had a high-protein caloric deficit of 42% that included refeed days. Resistance and cardiovascular exercise were each completed 4–5 times per week throughout preparation. The athlete self-reported weekly average bodyweight via an electronic survey. Additionally, at the beginning of preparation and once monthly afterward, body composition and psychological assessments (TFEQ-R18, SCQ-2, PHQ-SADS) were administered. Blood samples were drawn at the beginning of preparation and during the week of competition.

**Results**: Bodyweight decreased from the beginning of preparation to the week of competition (51.9 to 46.7 kg; Δ5.2 kg) and further post-competition (46.3 kg; Δ5.6 kg, R^2^ = 0.96), with a concurrent decrease in body fat percentage (17.2% to 12.3%; Δ4.9%) and fat-free mass (FFM) (42.9 to 41.0 kg; Δ1.9 kg) from beginning to competition week. Cortisol levels were elevated at the beginning of preparation (27.6 µg/dl) and elevated further during the week of competition (30.4 µg/dl). Total testosterone levels decreased from 46 pg/ml to 27 pg/ml (Δ19 pg/ml) within the same timeframe. It is possible that the low testosterone:cortisol ratio present at the start of preparation and continuing for the duration of preparation could be responsible for the loss of FFM. Emotional eating behaviors peaked post-competition (5/12), as did enthusiastic commitment (30/30) and sport enjoyment (25/25). Mild somatic (8/30) and depressive (5/27) symptoms manifested approximately one month prior to competition and remained elevated (7/30) post-competition. Depressive (7/27) and anxiety (5/21) symptoms all peaked post-competition but remained beneath the threshold for clinical significance.

**Conclusions**: To the authors’ knowledge, the present athlete’s loss of FFM is the first to be reported in a female bodybuilder. The athlete’s cortisol was also higher than other case reports, suggesting a hormonal consideration regarding FFM reduction during dieting phases in women. Moreover, the rise in symptom manifestation aligns with the peak in emotional eating behaviors post-competition and may be the first empirical documentation of what is anecdotally known as ‘post-show blues’.

**Acknowledgments**: The authors report no conflicts of interest. Funding for blood work was provided by Legion Athletics, Inc.

**The acute effects of a commercially available caffeinated and caffeine-free thermogenic dietary supplement on hunger and hemodynamic variables**

Kworweinski Lafontant^a^, Kara Phillips^a,b^, Jacob Broeckel^a^, Arielle Parks, Eric Velazquez^a^, Savannah Ericksen^a^, Indira Alur^a^, Gretchen Shelton^a^, Andrew Heath^a^, Cassandra Resler^a^, Scott Dankel^c^, and Bill I. Campbell^a^

^a^Exercise Science Program, College of Education, University of South Florida, Tampa, FL, 33,620, USA

^b^Department of Kinesiology, Nutrition, and Dietetics, University of Northern Colorado, Greeley, CO, 80,639, USA

^3^Health and Exercise Science Department, School of Nursing & Health Professions, RowanUniversity, Glassboro, NJ, 08028, USA

Corresponding author: Bill Campbell. bcampbell@usf.edu. 4202 E. Fowler Avenue, EDU 105, Tampa, FL, 33,620

**Background**: Caffeine is a typical active ingredient within multi-ingredient fat loss supplements due to its thermogenic properties. However, there has been a rise in the popularity of ”non-stimulant” or caffeine-free fat loss supplements in an attempt to circumvent caffeine-induced central nervous system (CNS) stimulation. Practically, the symptoms of an increased heart rate (HR), blood pressure (BP), and hunger perception are what users of non-stimulant supplements may seek to avoid. The purpose of this study was to examine the effects of a caffeinated and non-caffeinated commercially available fat loss supplement (Phoenix, Legion®; Phoenix Caffeine-Free, Legion®) on hunger, resting HR and BP variables in metabolically healthy adults.

**Methods**: Twenty-five male and female participants (age: 23 ± 4 yrs; height: 163 ± 10 cm; bodyweight: 69 ± 16 kg; BMI: 26 ± 5) completed this randomized, triple-blind, placebo-controlled cross-over study. Each laboratory visit was preceded by an overnight fast and 24-h exercise abstinence, and began with baseline assessments of height, weight, hunger, HR, and BP. Ensuingly, participants ingested three pills – with water – containing either the caffeinated (CAF; 200 mg), non-caffeinated (NCAF), or placebo (PL) fat loss supplement, and then repeated the hunger, HR, and BP assessments at 60-, 120-, and 180-minutes post-ingestion. Each visit followed the same protocol, with participants ingesting a different supplement condition during each visit, totaling three visits. Data were analyzed via a 3 × 4 (treatment x time) within-subjects repeated measures analysis of variance and paired samples t-tests for post-hoc analyses. Alpha was set at *p ≤*0.05.

**Results**: CAF significantly increased SBP at both the 120- and 180-minute time points compared to other conditions *(p <* 0.05). There was a significantly greater increase in DBP at the 60-minute timepoint in CAF compared to other conditions (*p* < 0.05). No differences in SBP nor DBP were present between NCAF and PL at any timepoint (*p >*0.05). There was a significant difference in HR at the 180-minute timepoint (*p* = 0.016), with a greater decrease in NCAF than in PL (*p* = 0.007), but no differences were present between CAF and any other condition (*p* >0.05). There was no difference in the change in hunger at the 60-minute or 180-minute timepoints (*p* >0.05). However, NCAF significantly decreased hunger at the 120-minute time point compared to other conditions *(p <* 0.05). No differences in hunger were present between CAF and PL at any timepoint (*p >*0.05).

**Conclusions**: The non-caffeinated supplement may function as a weight loss aid via hunger reduction. The hemodynamic data suggests that the non-caffeinated supplement does not stimulate the CNS, whereas the caffeinated supplement appears to do so.

**Acknowledgments**: This study was funded by Legion Athletics Inc. and the Florida High Tech Corridor.

**Sports Nutrition Knowledge and Status of NCAA Division II Athletes**

Elizabeth Newcomb-Yi^a^, Rachel Darr^a^*, Hillary Mellema^b^

^a^Department of Kinesiology, Saginaw Valley State University, University Center, MI; ^b^Department of Management and Marketing, Saginaw Valley State University, University Center, MI

*Corresponding author: rldarr@svsu.edu

**Background**: Individuals who partake in physical activity need to fuel their bodies adequately for optimal performance. Proper nutrition is essential for sufficient energy production required for athletics. Saginaw Valley State University (SVSU) athletes’ knowledge was obtained through a series of surveys. The purpose of this study was to utilize and apply concepts from nutrition to the student athlete population at SVSU, to decipher their knowledge, and establish a statistical need for future educational intervention to improve that knowledge.

**Methods**: Division II varsity athletes were asked 17-sports nutrition knowledge questions focusing on the topics of macronutrients, micronutrients, weight management, supplements, and hydration in an online survey. Participants provided information regarding the sources from which they obtained their nutritional knowledge. The total percentage correct was calculated for the sports nutrition portion of the survey.

**Results**: Sixty-two surveys with a mean score of 48.74% (8.29/17) were completed. Survey scores participants struggled with were ergogenic aids (15.87%), general daily macronutrient intake values (15.87%), and vitamin toxicity (20.63%). Athletes exhibited a greater knowledge of carbohydrate loading (71.43%), pre-exercise meals (82.54%), and supplementation (85.71%). A statistically significant crosstab (X^2 (1) =7.219, p = .007), most frequently reported top sources of information were registered dietitian/nutritionist (n = 29), athletic trainers (n = 25) and strength and conditioning specialists (n = 24) with athletes mostly recommending registered dietitian/nutritionists (n = 28), athletic trainers (n = 17) and then strength and conditioning specialist (n = 16) to others. Of 59 participants, 79.66% (n = 47) are aware that SVSU provides a registered dietitian for their athletes. Of those who are aware, 74.47% (n = 35) state that a registered dietitian/nutritionist is among their top three choices for nutrition information. An ANOVA with the overall composite nutrition knowledge score as the dependent variable with the following six groups: gender (t =0.545, p =0.588), age (t =1.088, p =0.282), years in sport (t =−3.254, p =0.002), understanding of nutrition (t =0.485, p =0.630), and using a coach (t =−1.900, p =0.063) or dietitian (t =−0.306, p =0.761) for nutrition information revealed statistical significance F(5, 42) =2.308, p =0.049.

**Conclusion**: Based on data analysis thus far, education intervention is warranted in the topic areas of ergogenic aids, basic macronutrient intake, and vitamins to improve the nutrition knowledge of athletes at the university. More years in sport showcased lower nutrition scores. When athletes obtain information from coaches vs. dietitians, the overall scores trend lower, however more participants are needed to confirm.

**Acknowledgments**: Funding provided by SVSU Undergraduate Student Led Research Grant.

**Time of Day Effects on Critical Force During an Intermittent Handgrip Fatiguing Protocol**

Justine M. Renziehausen^a^*, Modesto A. Lebron^a^, Joon-Hyuk Park^a^, Ethan C. Hill^a^, Adam J. Wells^a^, Jeffrey R. Stout^a^, and David H. Fukuda^a^

^a^University of Central Florida

*Corresponding author: Justine.Renziehausen@ucf.edu

**Background**: Previous literature suggests time of day affects maximal handgrip strength, however, assessing sustained handgrip efforts may be a more encompassing assessment for various populations. Measuring differences in maximal strength and fatigability throughout the day may assist both athletes and workers with physically demanding occupations to optimize performance and decrease the risk of injury. The purpose of this study was to determine the effects of time of day on critical force (F_Cr_), Y0 (peak force), performance variability (RMSE), impulse, MVC (maximal strength), and *k* (rate constant), during an intermittent handgrip fatiguing protocol.

**Methods**: Forty-one males (n =19) and females (n =22) between the ages of 18–55 years old (22.2 ± 4.3 years; 169.9 ± 8.4 cm; 76.8 ± 16.5 kg; 51.3 ± 7.2 MEQ score) completed this study. On visit 1, participants completed informed consent and questionnaires. On Visit 2, participants completed anthropometrics and familiarization trials. Visits 3, 4, and 5 consisted of temperature assessments, a maximal voluntary contraction (MVC) to determine warm-up intensity, a standardized warm-up, additional MVCs, and an intermittent handgrip fatiguing protocol, during which participants were asked to complete 60 repeated 4-second maximal isometric contractions with 1 second of rest for 5 minutes. Visits 3-5 were completed at 9:00 am, 2:00 pm, and 7:00 pm in a randomized order, with a minimum of 24 hours between visits. Two-way repeated measures ANOVAs [time (9:00 am, 2:00 pm, 7:00 pm) × sex (male, female)] were used to examine differences in all dependent variables at each time of the day between the sexes. Two-way repeated measures ANOVAs [peak/nadir × sex (male, female)] were conducted to examine differences in the highest and lowest performance values between the sexes.

**Results**: For the time of day analyses, there were no significant time × sex interactions for any dependent variable (p >.05); however, there was a significant main effect for time for MVC (p = 0.006) and impulse (p < 0.001) along with tympanic temperature (p < 0.001). For the peak/nadir analysis, there was a significant time × sex interactions for MVC (p < .001), F_Cr_ (p =.005), and RMSE (p =0.030), as well as the main effects for time (peak/nadir) for all dependent variables (p < 0.05).

**Conclusion**: In conclusion, MVC and impulse during an intermittent handgrip fatiguing protocol and temperature appear to be lower in the morning than in the evening, while significant differences in peaks and nadirs of all performance variables indicate individual differences throughout the day. Additionally, males may experience a greater magnitude of change for MVC, F_Cr_, and RMSE than females throughout the day.

**Acute Enzyme Co-Supplementation with Plant Protein Enhances Early Net Exposure to Amino Acids**

Sean M. Garvey^a^*, Kevin J. M. Paulussen^b^, Andrew T. Askow^b^, Max T. Deutz^b^, Colleen F. McKenna^b^, Takeshi M. Barnes^b^, Justin L. Guice^a^, Kelly M. Tinker^a^, Scott A. Paluska^b^, Alexander V. Ulanov^b^, Laura L. Bauer^b^, Ryan N. Dilger^b^, and Nicholas A. Burd^b^

^a^BIO-CAT, Inc., Troy, VA, USA; ^b^University of Illinois at Urbana-Champaign, Urbana, IL, USA

*Corresponding author: sgarvey@bio-cat.com

**Background**: Digestive enzymes are commonly consumed with food to improve macronutrient digestion and support gastrointestinal comfort. Dietary supplementation with microbial proteases, in particular, is an underutilized approach to enhance dietary protein digestion and postprandial blood amino acid (AA) concentrations. No studies to date have investigated the effect of microbial protease supplementation on less digestible protein sources (e.g. plant-based proteins). This clinical trial was designed to investigate the effects of co-ingestion of a mixture of three microbial proteases (P3) with pea protein on postprandial plasma AA concentrations in healthy adults.

**Methods**: The trade name of P3 is OPTIZIOME® P^3^ HYDROLYZER™, which is standardized to 31,875 HUT units of protease activity per 250 mg dose. In a randomized, double-blinded, crossover design, 24 healthy adults (12 females, 12 males; 27 ± 4 years; 173.2 ± 10.7 cm; 74.6 ± 11.2 kg; 24.8 ± 1.9 kg·m^2^) co-ingested 25 g pea protein isolate (20 g protein; 2.2 g fat) with either one dose of P3 or maltodextrin placebo (PLA). Blood samples were collected at baseline and repeatedly across 5 h after ingestion of pea protein with P3 or PLA to determine plasma AA concentrations via LC-MS-MS. Differences in AA concentrations over time were assessed using linear mixed effects models. Additionally, the net exposure to AA (incremental area under the curve, iAUC) was calculated for both early (0‒2 h) and total (0‒5 h) postprandial periods and compared using paired t-tests. The study was registered at ClinicalTrials.gov (NCT04821557).

**Results**: Average plasma total AA concentrations were significantly greater with P3 versus PLA during the 0‒5 h postprandial period (*P* = 0.010). There was a trend for elevated essential AAs (EAAs) across the 5 h period with P3 versus PLA (*P* = 0.099), but not for branched-chain AAs (BCAAs, *P* = 0.410) or leucine (LEU, *P* = 0.282). However, early net exposure (0‒2 h iAUC) to AAs was higher for P3 compared to PLA (*P* < 0.05 for all of total AA, EAA, BCAA, and LEU).

**Conclusions**: Microbial protease co-ingestion with pea protein isolate enhanced early net exposure to amino acids in healthy adults. Supplementation with microbial proteases is a safe and well-tolerated strategy to improve dietary protein digestion in healthy adults. This work provides a foundation for further exploration of fortifying foods and dietary supplements with microbial enzymes to improve protein digestibility and enhance early release of dietary amino acids into circulation.

**Acknowledgments**: Funding was provided by BIO-CAT, Inc.

**The Effect of Breakfast Consumption on Afternoon Resistance Training Performance in Habitual Breakfast Consumers and Non-Consumers**

Matthew T. Stratton^a,b^, Madelin R. Siedler^a^, Christian Rodriguez^a^, Patrick S. Harty^a,c^, Jake R. Boykin^a,d^, Dale S. Keith^a^, Jacob J. Green^a^, Sarah J. Whitea^a^, Ethan Tinoco^a^, Brielle DeHaven^a^, Trisha A. VanDusseldorp^e^ & Grant M. Tinsley^a^*

^a^Energy Balance & Body Composition Laboratory; Department of Kinesiology & Sport Management, Texas Tech University, Lubbock, TX, USA; ^b^Department of Health, Kinesiology and Sport, University of South Alabama, Mobile, AL, USA; ^c^Department of Kinesiology; College of Science, Technology, and Health; Lindenwood University, St. Charles, MO, USA; ^d^Department of Nutrition and Integrative Physiology, Florida State University, Tallahassee, FL, USA; ^e^Bonafide Health, LLC, JDS Therapeutics, Harrison, NY, USA

*Correspondence: grant.tinsley@ttu.edu

**Background**: Pre-exercise meal frequency is commonly believed to impact exercise performance, but little is known about its impact on resistance training performed later in the day once eating has resumed. Additionally, no previous investigations have examined the potential role of habitual breakfast consumption in these responses. Thus, the present study investigates the impact of breakfast consumption on afternoon resistance training performance and psychological measures in habitual breakfast consumers and non-consumers.

**Methods**: Thirty-nine resistance trained males (n =20) and females (n =19) who habitually consumed (≥ 5 days/wk; n =19) or did not consume (≥ 5 days/wk; n =20) breakfast completed three experimental trials. Following the establishment of single repetition maximums (1RM) on the first visit, on visits two and three participants completed a workout consisting of four sets of barbell back squat, bench press, and deadlift, utilizing 80% of their previously established 1RM either after consuming breakfast and lunch (B) or the same food only at lunch (BO). Number of repetitions, along with average and peak average concentric velocity (ACV), and power were measured for all repetitions throughout the session. Visual analog scales were used to assess feelings of fatigue, energy, focus, hunger, desire to eat, and fullness before and throughout each exercise session.

**Results**: Males performed significantly fewer repetitions on sets 2, 3, and 4 (*p*s < 0.014) for total repetitions when collapsed across all exercises. Differences were seen for barbell back squat on sets 2 and 4 (*p*s < 0.023) and set 4 for deadlift (*p* = 0.006), with no differences in repetition performance between the sexes for bench press. Males displayed reductions in average power across all sets and exercises except deadlift. No effect of habitual breakfast consumption was found.

**Conclusions**: These data suggest that alterations in meal frequency do not influence afternoon resistance training performance provided similar total pre-exercise nutritional intake.

**Acknowledgments**: Funding for this project was provided by Texas Tech University.

**Does Caffeine in Combination with L-Theanine Enhance Muscular Endurance While Mitigating Adverse Effects Often Associated with Caffeine Alone?**

Abigail Stack*, Jessica Roberts, Rebekah Seamans, Tomás Barrett

*Corresponding author: stackan2024@mountunion.edu

**Introduction**: Large doses of caffeine tend to result in greater improvements in muscular endurance. However, increasing caffeine levels can result in negative side effects like stress and jitteriness. L-theanine is an amino acid that is frequently added to caffeine containing products. It has been anecdotally attributed to mitigating caffeine’s adverse effects. Although caffeine and L-theanine are often combined, previous research examined their synergistic effects on non-exercise tasks. This study aims to investigate stress, jitteriness, and muscular endurance following the consumption of caffeine, L-theanine, L-theanine and caffeine combination, and a placebo, during exercise.

**Methods**: Ten recreationally active (≥150 min/week moderate activity) college-aged (18-24 y/o) students determined their max bench press (1-RM) and were familiarized with protocols. In the subsequent four sessions, participants received supplements (Caffeine (4 mg/kg bodyweight), L-Theanine (200 mg), Caffeine and L-Theanine (same dosages combined), or placebo) in randomized order with 72 hours (minimum) between sessions. Supplements were mixed into flavored water (8 oz). Upon arrival, participants completed a visual analog scale (VAS) and laid supine for 10 minutes (HRV analysis in the last 5 minutes). They consumed their assigned treatment and sat for 45 minutes. They completed a VAS, rested supine for 10 minutes (HRV analysis in the last 5 minutes), performed a 5-minute stationary bike warm-up (50 rpm), and performed one set of bench press repetitions to failure (RTF) at 60 bpm, using 60% of their 1-RM. Participants completed a post-exercise VAS, rested supine for 30 minutes (HRV analysis in the last 5 minutes), and completed the departure VAS.

**Results**: There was no significant difference in RTF between groups, F(3,39) = 1.173, p =0.334. The main effect of time on HRV was not statistically significant, Huynh-Feldt adjusted F(1.902,68.472) =1.263, p =0.289. The main effect of the group on HRV was not statistically significant, F(3,36) =2.516, p =0.074. The time X group interaction was not significant, Huynh-Feldt adjusted F(5.706,68.472) =1.943, p =0.089. The main effect of time on jitteriness was statistically significant, with sphericity assumed F(2,72) =19.413, p < 0.001. The main effect of the group on the jitteriness was not statistically significant, F(3,36) =0.538, p =0.659. The time X group interaction was not significant, sphericity assumed F(6,72) =0.815, p =0.562.

**Conclusions**: This study investigating stress, jitteriness, and muscular endurance following the consumption of caffeine, L-theanine, L-theanine and caffeine combination, or placebo, during exercise, concluded there was no significant difference in HRV, jitteriness, nor RTF between groups. Thus, within this study’s parameters, consumption of these supplements yields similar performance outcomes, self-perceived metrics of jitteriness, and biological stress metrics.

**The Acute Effects of Energy Drinks on Measures of Cognition and Muscular Activation in Female College Athletes Following Repeated Wingate Tests**

Edward Robinson*

Meredith College, Raleigh, NC

*Corresponding Author: ehrobinson@meredith.edu

In recent years, high-intensity workouts have increased in popularity. The appeal of this type of exercise for individuals is to provide an effective form of stimulus in a shorter amount of time. Another phenomenon affecting the exercise community is the growing popularity of the ubiquitous pre-exercise energy drinks which are sold in a variety of formula. Although the standard ingredient in energy drinks is usually caffeine, recent formulations have sought to include a variety of ingredients to boost the feeling of alertness and increase the physical performance effects from these beverages. Recently, supplementation companies have begun to market energy drinks as a form of pre-workout supplementation. To date, the majority of studies examining this topic have studied either energy drinks containing traditional formulas (caffeine) vs placebo or sport-focused energy drinks (with creatine, taurine, etc.) vs placebo, but have not combined them in a single study. Additionally, none have evaluated the effects of these energy drinks on the neural activation of muscle during an exercise event.

**Methods**: Ten college athletes performed repeated Wingate tests on three separate occasions. Prior to exercise, each individual was asked to drink either a sports-focused energy drink, popular commercial energy drink, or a placebo an hour prior to exercise. Supplementation was double-blinded and the order randomized. Prior to and immediately post exercise, individuals performed a Stroop test and a serial subtraction test to assess changes in cognitive ability. Electrodes were placed on the vastus lateralis and the rectus femoris to assess muscle activation during the Wingate tests.

**Results**: Paired sample tests revealed a significant difference between pre- and post-exercise scores on the Stroop test (p =0.044) during the placebo trial but no significant differences for any other measures of cognition. Repeated ANOVA measurements revealed a significant difference in average power for the vastus lateralis during the Wingate tests for the sports-focused energy drink, but not for the traditional energy drink or the placebo. In the rectus femoris, significant differences were seen in muscular activation in both sport-focused and placebo trials but not in traditional. Maximal EMG only demonstrated a significant difference in the rectus femoris muscle during the placebo trial.

**Conclusions**: In this study, energy drinks did provide evidence of aiding cognitive ability after a repeated sprint effort in female college athletes. However, muscle activation decreased in both sports-specific and placebo groups in subsequent efforts, suggesting that fatigue may have occurred. Further research is recommended.

**Effect of Ultra-Endurance Running on Athlete Heart Rate and Blood Pressure**

Steven B. Hammer^a^, Fred R. Strale^b^, Timothy B. Williams^c^*, Shantele Kemp Van Ee^c^, James Agnew^d^

^a^Associate Professor, Indian River State College, Fort Pierce, Florida; ^b^Adjunct Professor, Indian River State College, Fort Pierce, Florida; ^c^OMS-III, Nova Southeastern Kiran C. Patel College of Medicine, Fort Lauderdale, Florida; ^d^Adjunct Professor, Indian River State College, Fort Pierce, Florida

*Corresponding author: tbwilliamsdo@gmail.com

**Background**: While the effects of exercise on the cardiovascular system are well documented, ultra-endurance sports, such as marathons, triathlons, and Ironman competitions are relatively modern and are growing in popularity. While brief, high-intensity exercise stimulates the sympathetic nervous system, including increases in heart rate and blood pressure, fluid, and electrolyte depletion, which commonly occurs in ultra-endurance events, may contribute to decreases in overall blood pressure after the race. If decompensation of the autonomic safety net occurs, orthostatic hypotension as a result of fluid loss during an event may cause fatigue, dizziness, syncope, or collapse.

**Methods**: We observed blood pressure and heart rate changes in subjects before and after marathon activity in both supine and standing positions over multiple races of variant length and terrain from 50 kilometers to 240 kilometers from 2013 to 2018. Post-race measurements were observed 2-12 hours after completion time. In addition to pre- and post-race measurements, positional post-race blood pressures and heart rates were analyzed for orthostatic trends.

**Results**: Of 217 pre-race participants, 140 race completions were observed. Pre- and post-race HR and BP measurements were recorded. The mean age of the runners was 43.8, with an average BMI of 21.2. The mean systolic blood pressure increased pre- to post-race standing was 21 mmHg, while the mean diastolic rise was 13 mmHg. While supine, there was a 15 mmHg systolic BP increase pre- to post-race and a 7 mmHg diastolic rise. Post-race supine to standing average blood pressure change was insignificant. In the supine position, the mean heart rate increase after the race was 20 beats per minute (bpm) and 27 bpm while standing. After the race, the average increase in HR supine to standing was 15 bpm.

**Conclusion**: Systolic blood pressure changed notably in comparison to diastolic pressures, likely due to the increase in stroke volume associated with the sympathetic nervous system response during exercise. Heart rate values also climbed as a result of exercise stress in the setting of catecholamine release, and the combined influence contributed to increased cardiac output despite water and electrolyte loss during the event. Post-race, no trends of orthostatic hypotension were noted either with HR or BP when rising from a supine position. The contribution of fluid intake during the race and compensatory mechanisms under neural control requires further study.

**A Steady-State Simulation of the Pharmacokinetics of d9-Caffeine Versus Regular Caffeine in Humans and Recommended Daily Consumption Level**

Bradford C. Sippy*, Paul M. Tarantino, Mary M. Sherman

Lennham Pharmaceuticals, Inc. Concord, MA

*Corresponding author: brad.sippy@lennham.com

**Background**: d9-Caffeine is being proposed as a long-acting substitute for regular caffeine in food and beverages. A single dose pharmacokinetic study in humans was completed showing that d9-caffeine exhibited a similar T_max_, a higher C_max_ and substantially higher AUC_0-48h_ than caffeine. A steady-state model was used to determine if the results shown in the single-dose comparative study were consistent when the two products were administered daily, which is the predominant consumption pattern for caffeine-containing products.

**Methods**: A steady-state analysis of the exposure of caffeine relative to d9-caffeine in humans was constructed based on the results of the single-dose cross-over comparison and was consistent with industry best practices for the construction of such models from single-dose studies. As good dose proportionality was observed for both d9-caffeine and caffeine, the dose normalized steady-state values of each moiety administered once every 24 hours were also compared.

**Results**: The steady-state model demonstrated results consistent with the single-dose study, whereby the slower metabolism of d9-caffeine results in a prolonged exposure to d9-caffeine relative to caffeine. d9-Caffeine demonstrated an accumulation factor ranging from 2.23-2.56 compared to caffeine’s accumulation of 1.03-1.07. At steady state, d9-caffeine was predicted to have multiple exposures to caffeine, based on C_max-ss_ and AUC_0-24_, ranging from 2.73-3.49, compared to AUC_0-48_ multiples ranging from 4.35-4.88 demonstrated in the single-dose study comparison.

**Conclusions**: At steady state, d9-caffeine exhibits a higher C_max_ and higher AUC_0-24h_ than caffeine and a modest accumulation factor. The multiples of exposure at steady state between d9-caffeine and caffeine are similar to those seen in the single-dose study, even when accounting for repeat daily dosing of both products. The long-lasting nature of d9-caffeine and its corresponding multiples of caffeine exposure should be factored into any recommendations regarding the safe daily limit of d9-caffeine. These results support an initial Generally Recognized as Safe (GRAS) level of approximately 100 mg of d9-caffeine, which would be comparable to the generally accepted current 400 mg GRAS limit of regular caffeine.

**Acknowledgments**: B. Sippy is the founder of Lennham Pharmaceuticals and owns stock in the company. P Tarantino and M Sherman are consultants to Lennham Pharmaceuticals. This research was supported by Lennham Pharmaceuticals.

**The Correlation Between Advanced Glycation End Products and Cardiorespiratory Fitness**

Maximus Betscakos^a^*, Taylor Catlett^a^

^a^Department of Exercise, Sport, and Nutrition Sciences, University of Mount Union, Alliance, OH, 44,601, USA

*Corresponding author: betscamg2023@mountunion.edu

**Background**: Cardiovascular Disease (CVD) is the leading cause of death worldwide, driven by risk-factors, such as sedentary lifestyles and/or nutritionally poor diets. Advanced glycation end-products (AGE) are sugars that bind to lipoproteins within the body over a lifetime of eating a highly processed diet, while cardiorespiratory fitness (CRF) is a measure of one’s aerobic capacity. Literature has shown that AGEs and CRFs can be utilized to evaluate the risk of CVD.^[1,2]^ Therefore, further evaluation on the relationship between AGEs and CRFs is warranted. If a correlation exists, then elevated levels of AGEs within a patient may indicate a low level of CRF based on the shared use for determining levels of CVD risk that both AGEs and CRF possess. We hypothesized that there would be a negative correlation between AGEs and CRFs.

**Methods**: Forty Caucasian general population male and female adults (18+) completed three separate assessments throughout this study: a BodPod assessment; AGE assessment; and a Cooper 12-minute aerobic assessment. Participants completed the BodPod (COSMED) assessment following the COSMED protocols. Participants then completed the AGE assessment utilizing the AGE Reader mu-Connect (Diagnoptics Technologies, Groningen, Netherlands) to obtain measurements. Finally, participants arrived at the indoor track to complete the Cooper 12-minute aerobic assessment following the Cooper Protocol.^[3]^

**Results**: The correlation between AGEs and CRFs for the total population showed no statistical significance (r =−0.224; p =0.164; Table 1). The correlation between AGEs and CRFs for females also showed no statistical significance (r =−0.285; p =0.223; Table 2). However, the correlation between AGEs and CRF for males indicated statistical significance (r =−0.457; p =0.043; Table 3).

**Conclusion**: Within this study, statistical significance between AGEs and CRFs only existed in the male population, while the female and total populations did not contain statistical significance between AGEs and CRFs. We hypothesize that the results for total and female populations may be due to not utilizing a maximal aerobic assessment, which may have allowed effort to play a role in the results. In addition, the AGE reader cannot measure dark skin tones due to the high levels of melanin within the skin, and thus our study only examined within a fairer skin population. With this, future research should implement the use of maximal aerobic assessments to prevent the effort effect, AGE blood testing to include all populations, as well as a food log analysis to provide a better perspective regarding AGE results.

**Acknowledgments**: Thank you to Dr. Ronald Mendel and all the participants in our study.

**Does Range of Motion or Angle Influence Hyperemia and Oxygen Concentration of the Pectoralis Major During Bench Press?**

Daniel E. Newmire*, Michelle Eisenman, Ronald L. Snarr

Exercise Biochemistry and Physiology Lab; Department of Kinesiology; Texas A&M University-Corpus Christi; Corpus Christi, TX USA

*Corresponding author: daniel.newmire@tamucc.edu

**Background**: Fluid accumulation (i.e. hyperemia) within skeletal muscle post-resistance training has been shown to be associated with increases in muscle growth. While hyperemia can be influenced by multiple factors, such as range of motion (ROM) and angle, the combination of these factors in post-exercise muscle swelling has yet to be examined. Therefore, the purpose of this study was to investigate the effect of bench press ROM and angle on regional hyperemia (CSA%), oxygen (SmO_2_), and hemoglobin (tHb) concentration of the pectoralis major (PM).

**Methods**: Ten subjects (6 M/4 F; age:23.8 ± 2.3y; Ht:170.1 ± 3.5 cm; Wt:72.96 ± 5.6 kg) completed the eight-week study consisting of four bouts of strength testing (~75-80% one-repetition maximum [1RM]) with subsequent ROM bouts. Each bout was randomized to full (fROM) or partial (pROM) ROM during flat (FB) and incline (IB) (45°) bench press using a Smith Machine (SM). For each condition, participants completed three sets of bench press until volitional concentric failure. For the pROM bouts, the SM barbell was fitted with an EliteFTS™ ‘*shoulder saver pad*’. The vertical distance (cm) of the barbell was measured with a linear transducer. Pre-bout and 3-min post-bout blood lactate (BLa) were collected, via finger prick, using a lactate analyzer. A near-infrared spectroscopy unit was placed on the subject’s left side approximately mid-muscle belly of the PM (~25%) to assess SmO_2_ and tHb concentrations. Cross-sectional area (CSA) was measured using panoramic capability ultrasound at pre-bout (Pre), immediate post-bout (IP), and 24-hr post-ROM bout of the right-side PM at 10%, 25%, 40%, 50%, and 60% distal of the suprasternal notch in males. Multiple analyses of variance were used to assess change (Δ) in regional hyperemia, load volume (kg⋅sets⋅repetitions), volume (sets⋅repetitions), 1RM intensity, BLa, SmO_2,_ and tHb concentrations.

**Results**: Preliminary results showed no effect (n =4; η_p_^2^ =0.38) of condition (ROM and angle) on regional hyperemia (ΔCSA%), BLa (n =9; ω^2^ =0.72), ΔSmO_2,_ or ΔtHb (n =3; ω^2^ =0.20) concentrations. The highest load volume was found in the pROM-FB condition (4,842 ± 1,038 kg); with the lowest load volume (3,030 ± 555.9 kg; n =10; ω^2^ =0.84), volume (n =10; ω^2^ =0.60), and vertical distance (n =6; ω^2^ =0.54) in the fROM-IB condition. No difference was found in 1RM intensity between conditions (n =10; ω^2^ =0.60).

**Conclusion**: The preliminary results show that pROM-FB had the highest load volume concurrent with the shortest distance. In contrast to previous work showing the effect of bench press angle on PM regional amplitude, angle nor ROM had an impact on post-exercise regional blood flow or oxygen concentration of the PM.

**The Effects of an Amino Acid-Containing Electrolyte Beverage on 5-Kilometer Performance, Blood Electrolytes, and Post-Exercise Cramping Versus a Conventional Carbohydrate-Electrolyte Sports Beverage and Water**

Mason C. McIntosh^a^, Bradley A. Ruple^a^, Nicholas J. Kontos^a^, Madison L. Mattingly^a^, Christopher M. Lockwood^b,c^, Michael D. Roberts^a^*

^a^School of Kinesiology, Nutrabolt Applied and Molecular Sciences Laboratory, Auburn University, Auburn, AL USA; ^b^Nutrabolt, Austin, TX, USA; ^c^Dr Chris Lockwood LLC, Casper, WY, USA

*Corresponding author: mdr0024@auburn.edu

**Background**: The purpose of this study was to examine the acute effects of a multi-ingredient pre-workout supplement (MIPS, XTEND® Healthy Hydration) on 5-kilometer (5-km) time trial performance compared to a carbohydrate-electrolyte beverage (CE, GATORADE® Thirst Quencher) and distilled water (W).

**Methods**: Twenty healthy participants (10 men and 10 women, 20-35 years old, BMI ≤29 kg/m^2^, recreationally active) completed this study in a single-blinded, randomized and controlled, crossover fashion. For the first visit (T1), participants performed testing for urinary specific gravity (USG), a maximal aerobic treadmill test, and a familiarization treadmill run. The following week (T2), participants reported to the laboratory after a 12-14-hr overnight fast and completed a testing battery that included (T2-1) USG, cramp and mood questionnaires, heart rate (HR) and blood pressure (BP) measurements, finger sticks to measure blood-electrolytes, and nude body mass assessments. Participants were then provided 16 fl. oz. of either CE, W, or MIPS, in randomized order, and rested for 45 minutes. Following the rest (T2-2), participants completed USG, questionnaires, HR and BP measures, and a finger stick. Participants then performed a 5-km time trial on a treadmill with metabolic testing using indirect calorimetry. Immediately after the time trial (T2-3), participants completed the questionnaires, HR and BP measures, a finger stick, and a nude mass assessment. After 60 minutes (T2-4), participants’ HR and BP were measured, and a finger stick was completed. After another 60 minutes (T2-5), participants’ USG was measured, they completed two questionnaires, HR and BP were measured, and a finger stick and body composition was collected. Participants returned to the laboratory approximately one (T3) and two weeks (T4) following T1 to replicate testing and consume the other two drinks.

**Results**: No significant differences occurred between the conditions for 5-km time trial completion, indirect calorimetry outcomes during 5-km time trials, USG, or nude mass measurements (p >0.05 for all relevant statistical tests). However, the blood potassium and the sodium/potassium ratio displayed significant interactions (p < 0.05), and post hoc testing indicated that these values were better maintained in the MIPS versus other conditions. Post-exercise cramp prevalence was greater in the CE (p < 0.05) and trended higher with W (p =0.083) compared to the MIPS condition. Post-exercise cramp severity was also elevated with the W and CE beverages (p < 0.05) but not the MIPS (p =0.211).

**Conclusions**: MIPS did not affect 5-km time trial performance but exhibited favorable effects on blood electrolyte and post-exercise cramp outcomes compared to the CE and W drinks.

**Acknowledgments**: This study was supported by a contract awarded to the laboratory of M.D. Roberts at Auburn University by Nutrabolt (Austin, TX, USA). C.M. Lockwood is the VP of Scientific Affairs at Nutrabolt. He was involved in the study design but did not partake in data collection or statistical analysis. M.C. McIntosh was fully supported through a research fellowship from the National Institutes of Health (T32GM141739).

**Combination of Curcumin and Alpha-Lipoic Acid is Safe and Effective in Reducing Bodyweight and BMI in Overweight Adults: A Double-Blind Placebo-Controlled Clinical Study**

Sidney Abou Sawan^a^*, Venkata Krishnaraju Alluri^b^, Raza Bashir^a^

^a^1Iovate Health Sciences International Inc, Oakville, ON, Canada; ^b^Laila Nutraceuticals, Vijayawada, AP, India.

*Corresponding author: sidney.abousawan@iovate.com

**Background**: Natural compounds curcumin and alpha-lipoic acid have potential benefits in weight management in preclinical and clinical studies. Curcumin reduces inflammation and improves insulin sensitivity, while alpha-lipoic acid increases energy expenditure, mitochondrial function, and glucose uptake. We have previously demonstrated that combining these compounds may have a synergistic effect in promoting weight loss in mice. In this study, we aimed to evaluate the safety and efficacy of curcumin and alpha-lipoic acid in weight management in overweight humans.

**Methods**: In a double-blind, placebo-controlled clinical study, 100 healthy overweight-obese men and women (aged 21 – 45) were randomly assigned to consume 150 mg curcumin and 400 mg alpha-lipoic acid (CURLA; n = 50) or placebo (PLA; n = 50) twice daily for 16 weeks, alongside an 1800 calorie diet and 30 minutes of walking per day. Changes in body weight (primary outcome), lean body mass (LBM) and fat mass (FM) (DEXA), waist circumference, lipid profiles (LDL, HDL, VLDL, triglycerides, total cholesterol), appetite (Visual Analog Scale) and mood (Profile of Mood States-Short Form) was assessed pre and post intervention.

**Results**: After 16 weeks, both groups CURLA (n = 42) and PLA (n = 40) reduced bodyweight, BMI, and waist circumference (all P < 0.001). However, CURLA showed greater reductions in bodyweight (~93%; 5.18 ± 3.01 kg vs 2.68 ± 1.7 kg), BMI (~91%; 1.91 ± 1.12 kg/m^2^ vs 1.0 ± 0.63 kg/m^2^), and waist circumference (~33%; 3.52 ± 1.9 cm vs 2.64 ± 2.1 cm) compared to PLA (P ≤ 0.036). Both groups showed reductions in LBM, FM, and appetite (all P < 0.01) with no differences between the two groups (P ≥ 0.52). After 16 weeks, lipid profiles and mood remained stable within (P ≥ 0.15) and between the two groups (P ≥ 0.66).

**Conclusions**: The combination of curcumin and alpha-lipoic acid, when consumed twice daily for 16 weeks alongside an 1800 calorie diet and exercise, resulted in greater reductions in bodyweight, BMI, and waist circumference compared to the placebo group. The intervention was well tolerated and did not have any adverse effects. These findings suggest that curcumin and alpha-lipoic acid are effective natural compounds for weight management in overweight individuals.

**Acknowledgments**: Iovate Health Sciences International Inc sponsored the study, which was conducted by Laila Nutraceuticals.

**Pharmacokinetic Analyses of Food and Non-Food-Derived Multi-Vitamin/Mineral Formulations I: Serum Changes**

Joungbo Ko, Choongsung Yoo, Dante Xing, Drew E. Gonzalez, Victoria Jenkins, Broderick Dickerson, Megan Leonard, Kay Nottingham, Jacob Kendra, Ryan Sowinski, Christopher J. Rasmussen, Richard B. Kreider*

Exercise & Sport Nutrition Lab, Texas A&M University, College Station, TX 77843, USA

*Corresponding Author: rbkreider@tamu.edu

**Background**: The International Society of Sports Nutrition recommends that active individuals and athletes ingest a daily multivitamin and mineral supplement (MVM) to meet micronutrient needs. For this reason, ingestion of a daily multivitamin is a common practice to promote general health among athletes. However, there has been a debate about whether organic vitamins and minerals are more bioavailable than non-food sources of vitamins and minerals. This study compared the pharmacokinetic profiles of food-derived (FD) MVM supplements to non-food-derived (N-FD) MVM supplements.

**Methods**: In a randomized, crossover, and double-blind manner, 34 men and women fasted for 12 hours, donated a blood sample, and ingested a standardized breakfast (Nature Valley Oats ‘N Honey crunch granola bar, General Mills, Inc, Minneapolis, MN, USA) with either an N-FD MVM supplement (Nutraceutical Corp., Salt Lake City, UT, USA) or a food-derived (FD) MVM supplement (Garden of Life mykind Organics, Women’s Formula, West Palm Beach, FL, USA) supplement. Blood samples were taken 2, 4, and 6 hours after ingesting the meal and supplement. Participants observed a 7 to 14-day washout period and repeated the experiment while consuming the remaining assigned treatment. Samples were analyzed by Heartland Assays LLC (Ames, IA, USA) using standard analytic procedures. Data were analyzed by a General Linear Model (GLM) univariate analyses with repeated measures and mean changes from a baseline with 95% Confidence Intervals (CI).

**Results**: Multivariate analysis revealed a significant time (*p* < 0.001, $\eta_{p}^{2}$ = 0.442, large effect) with no significant treatment × time effects (*p* = 0.331, $\eta_{p}^{2}$ = 0.039, weak effect) in changes in serum vitamin A, E, B_12_, and C and calcium, iron, or magnesium levels. Univariate analysis revealed no significant differences in vitamin A, E, and B_12_ levels or calcium, iron, or magnesium levels. There is a significant interaction effect (*p* = 0.042, $\eta_{p}^{2}$ = 0.046, weak to moderate effect) on vitamin C, with changes in vitamin C higher in the FD MVN after 2 hours but not significantly different than N-FD MVN at 4 or 6 hours.

**Conclusions**: Ingesting a food-derived MVN supplement does not appear to significantly improve the absorption of water or fat-soluble vitamins or minerals studied into the blood or the rate of clearance out of the blood compared to a non-food-derived MVM.

**Acknowledgments**: This study was funded as a grant to the Exercise & Sport Nutrition Lab at Texas A&M University by Nutraceutical/Better Being Co. (Salt Lake City, NV, USA).

**Sex Differences in Maximum Force and Neuromuscular Excitation Following Unilateral and Bilateral Fatiguing Tasks**

Balea J. Schumacher, John Paul V. Anders, Brady R. Fishpaw,

Department of Human Sciences, The Ohio State University, Columbus, OH, USA

*Corresponding author: schumacher.261@osu.edu

**Background**: Previous studies have demonstrated sex differences in exercise-induced fatigability, however, the mechanisms remain unclear. The purpose of the present study was to examine sex differences in maximum force and neuromuscular function following unilateral (UL) and bilateral (BL) fatiguing tasks.

**Methods**: Twelve participants (Males: *n* =6; Females *n* =6; age =23.3 ± 2.5 yrs; height =168.7 ± 7.4 cm; body mass =68.8 ± 11.4 kg) performed left leg UL maximal voluntary isometric contractions (MVICs) at 157°s^−1^ knee flexion before and after body weight squats to failure, performed as either UL or BL in random order on two separate days (48-hours between). During each MVIC, maximum force and electromyographic amplitude (EMG AMP) and mean power frequency (EMG MPF) from the left leg vastus lateralis were recorded. Separate 2(Time: Pre- vs. Post-fatigue)x2(Fatigue: UL vs. BL)x2(Sex: Male vs. Female) mixed-factorial ANOVAs were used to examine maximum force, EMG AMP, and EMG MPF. Follow-up *post-hoc* Bonferroni-corrected pairwise comparisons were conducted when appropriate. Intraclass correlations (ICCs) were determined from pre-fatigue MVICs from each visit for all parameters with a 2,1 model. An alpha value of *p* < 0.05 was considered statistically significant, and effect sizes were reported as partial eta-squared (η^2^_p_) and Cohen’s *d* for significant ANOVAs and pairwise comparisons, respectively.

**Results**: The ICC for maximum force (0.93) was considered excellent and the ICCs for EMG AMP (0.78) and EMG MPF (0.89) were considered good. For maximum force, there were no significant 3- or 2-way interactions, but a significant main effect for Time (*p* = 0.006, η^2^_p_ =0.544), with *post-hoc* analysis demonstrating Pre-fatigue (1561.9 ± 343.3 N) was significantly greater than Post-fatigue (1348.7 ± 353.7 N, *d* =0.29). For EMG AMP, there were no significant 3- or 2-way interactions, but significant main effects for Sex (*p* = 0.027, η^2^_p_ =0.402) and Time (*p* = 0.038, η^2^_p_ =0.364). The *post-hoc* analysis for sex demonstrated Males (0.82 ± 0.21 μV) were significantly greater than Females (0.50 ± 0.21 μV, *d* =1.52). The *post-hoc* analysis for Time demonstrated Pre-fatigue (0.63 ± 0.21 μV) was significantly less than Post-fatigue (0.70 ± 0.23 μV, *d* =0.34). For EMG MPF, there were no significant 3- and 2-way interactions, but a significant main effect for Time (*p* = 0.021, η^2^_p_ =0.426), with *post-hoc* analysis demonstrating Pre-fatigue (111.1 ± 17.8 Hz) was significantly greater than Post-fatigue (100.8 ± 15.6 Hz, *d* =0.62).

**Conclusion**: The results demonstrated that the participants exhibited excitation-contraction coupling failure, characterized by a decline in maximum force with an increase in neuromuscular excitation. These findings also suggested that the participants exhibited peripheral fatigue associated with the buildup of metabolic byproducts. While the males exhibited greater muscle excitation than the females, both sexes.

**Diets of Female DII Collegiate Volleyball Players as Viewed Through the Lens of ISSN Position Stands**

Holli Bragg^a^, Greg Popovich^a^, Kristy Henson^b^

^a^School of Exercise Science & Athletic Training, West Virginia Wesleyan College, Buckhannon, WV, 26,201 USA; ^b^School of Science & Technology, Fairmont State University, Fairmont, WV, 26,554 USA

Corresponding author: bragg.hr.2019@wvwc.edu

**Background**: This study provides insight into the dietary habits of in-season DII female collegiate volleyball players from two different universities in West Virginia through comparison to ISSN position stands. Tracking dietary intake of the macronutrients is important for athletes to perform and recover optimally.

**Methods**: Participants (n =16) were current DII female volleyball players over the age of 18. Participants tracked their normal daily food intake for five days using the USDA’s MyPlate meal tracking application. Participants then downloaded and submitted their data for anonymization and analysis. Findings were compared to the ISSN’s relevant position stands on protein, creatine, caffeine, meal frequency, nutrient timing, and energy drinks. The findings were compared between West Virginia Wesleyan College and Fairmont State University volleyball female athletes.

**Results**: The average caloric intake was 1,922.18 kilocalories (SD 792.7 kcal) per day. The daily macronutrient intake averages were as follows: fat 77.58 g (SD 33.12 g), carbohydrates 218.42 g (SD 117.26 g) and protein 68.24 g (SD 26.01 g). The fat intake average of 77.58 g fell just below the recommended daily amount of 83.33 (30% of 2,500 kcal per day). The carbohydrate intake average of 218.42 g fell within the recommended range of 5-12 g/kg/day. The protein intake average of 68.24 g fell below the recommended range of 0.8 g/kg/bodyweight. The average height of participants was five feet seven, and the average age was 20-years old. One athlete reported using creatine at an infrequent rate. One hundred percent of athletes reported using caffeinated beverages multiple times a day, including consuming energy drinks and preworkout. Fifty percent of the athletes reported supplementing themselves with vitamins. Most athletes did not record their food daily, which affected data analysis.

**Conclusions**: The average caloric intake of these athletes was found to be lower than the recommended ranges. The average range fell to 577.82 kcal, below the recommended 2,500 kcal per day. This is attributed to the lower range of protein intake. Fat and carbohydrate averages fell within the recommended ranges. Per the ISSN, the minimum recommended intake of calories for athletes is 2,500 kcal/day. The recommended intake ranges are 5-12 g/kg/per day and 1.4-2.0 g/kg/day for carbohydrates and protein. The recommended fat intake was 30% the total calories per day, and the players’ fat intake was appropriate heading toward the higher end of the fat intake range. The lack of athletes reporting daily dietary intake affected the average rates. Giving the participants an incentive or having coaches be more involved may increase compliance with data collection in the future.

**Acknowledgments**: This work was supported by the National Science Foundation through the First2 Network (https://first2network.org) National Science Foundation INCLUDES Alliance under NSF cooperative agreements HRD-1834569, HRD-1834575, HRD-1834586, HRD-1834595, HRD-1834601

**Effects of the Probiotic *Lactobacillus Acidophilus* on Acute, Subacute, and Sustained Lactose Tolerance in Healthy Adults**

Jaci Davis^a^*, Christine Florez^a^, Jessica Prather^a^, Javier Zaragoza^a^, Mandy Parra^a^, Grant Tinsley^b^, Christopher M. Lockwood^c^, Lem Taylor^a,d^

^a^Human Performance Laboratory, University of Mary Hardin-Baylor, Belton, TX, USA; ^b^Energy Balance & Body Composition Laboratory, Texas Tech University, Belton, TX, USA; ^c^Dr Chris Lockwood, LLC, Casper, WY, USA; ^d^Doctor of Physical Therapy Program, University of Mary Hardin-Baylor, Belton, TX, USA

*Corresponding author: jaci.davis@umbh.edu

**Background**: The purpose of this study was to determine if supplementation of lactobacillus acidophilus MPH734 (Lacto-Freedom^TM^, LF) for one week would affect acute, subacute, and post-treatment discontinuation (30 day) lactose metabolism, gastrointestinal symptoms, and clinical safety markers versus placebo. Many healthy adults avoid consuming dairy due to the potential presence of lactose and associated adverse gastrointestinal side effects.

**Methods**: Fifty-five healthy adult male and female (23.3 ± 5.8 yrs, 167.4 cm ± 10.5 cm, 77.5 kg ± 20.4 kg, 27.4 kg/m^2 ± 6.2 kg/m^2; mean ± SD) participants who regularly avoid products that contain dairy or lactose ingredients were randomly assigned in a double-blind manner to low (LFlow) or high-dose probiotic (LFhigh), or placebo treatment. Blood safety panels and ratings of diarrhea, gas, bloating, and abdominal pain were assessed, following weekly 25-gram lactose challenges for 4 weeks. Variables were assessed in the laboratory at days 0, 7, and 37. Data were analyzed using repeated measures analysis of variance, with Tukey post hoc pairwise comparisons when needed. The statistical significance was *p* < 0.05.

**Results**: For the weekly at-home testing, a significant treatment by time (pre/post challenge) interaction was observed for ratings of diarrhea (*p* = 0.03). Follow-up analysis indicated that ratings of diarrhea were higher in the placebo than LFhigh group following the lactose challenge (1.1 ± 0.3 [mean ± SE], *p* = 0.04), with a trend of higher diarrhea in the placebo group as compared to LFlow (0.9 ± 0.3; *p* = 0.08). Additionally, diarrhea ratings increased in the placebo group following the lactose challenge (1.1 ± 0.2, *p* = 0.001), but not in LFhigh (0.2 ± 0.3, *p* = 0.98) or LFlow (0.4 ± 0.2, *p* = 0.51). No treatment by time interactions were observed for at-home ratings of gas (*p* = 0.58), bloating (*p* = 0.50), or abdominal pain (*p* = 0.67) nor laboratory assessments of diarrhea (*p* = 0.20), gas (*p* = 0.36), bloating (*p* = 0.45), or abdominal pain (*p* = 0.44). Blood safety markers on CMP and CBC panels were not influenced (p >0.05) by ingestion of the treatments.

**Conclusions**: The primary conclusion of this study was that the rating of diarrhea was higher in the placebo vs. LFhigh and LFlow groups during weekly at-home testing. There were no significant differences between groups for gas, bloating, or abdominal pain. The data concludes that individuals that regularly avoid products that contain dairy may observe lower symptoms of diarrhea when taking either a low or high dose L. acidophilus MPH734 probiotic. The supplement also appears safe for ingestion based on blood safety profiles.

**Acknowledgments**: This study was funded by Manzo Pharmaceuticals, LLC

**Exploring the Novel Concept of Intranasal Administration of Creatine**

Connor Hollen, Greg E Popovich

School of Exercise Science & Athletic Training, West Virginia Wesleyan College, Buckhannon, WV, 26,201 USA

Corresponding author: hollen.ca.2020@wvwc.edu

**Background**: Creatine dysregulation has been shown to be evident in a number of psychiatric and neurocognitive conditions. In recent research, oral creatine monohydrate supplementation has shown improvement in memory capacity in older individuals, and in other research has been shown to reduce the cognitive symptoms of sleep deprivation. Animal research suggests that creatine supplementation may aid neuroplasticity during recovery from addiction. This growing body of evidence gives rise to the hypotheses that creatine could be a viable treatment for a wide range of conditions, such as dementia, depression, and concussion. Using an intranasal route of administration (ROA) of creatine could be a plausible means of efficiently dosing creatine to achieve absorption across the blood-brain barrier while targeting preferential delivery to the brain.

**Methods**: We searched the following databases to identify published studies on the intranasal ROA for creatine: PubMed, SciTech Premium Collection, CINAHL, and ACS Journals. These same databases provided reference material pertinent to comparisons between molecules regarding molecular weight and solubility.

**Results**: We did not identify any articles that addressed intranasal ROA of creatine. We were able to make a number of meaningful comparisons to molecules that are successfully absorbed across nasal epithelium. For example, insulin, consisting of 51 amino acids and possessing a molecular weight of 5,778 g/mol is now being administered as a nasal spray. In contrast, creatine, an amino acid derivative, is a far smaller individual molecule with a molecular weight of 151.1 g/mol. Cocaine, at 303.4 g/mol, is a tropane alkaloid that is known to be administered and absorbed intranasally. In comparison, creatine is more soluble at 14 g/L at 20°C, while cocaine is only 1,800 mg/L at 22°C.

**Conclusion**: For a variety of reasons including tissue targeting, avoidance of gastrointestinal degradation and minimizing first-pass hepatic metabolism, finding an alternative for oral medication is an ongoing project worldwide where investigators are finding new methods to administer medication to patients. We find a sufficient rationale based on relatively favorable molecular weight and solubility to suggest that research using a mammalian tissue model is warranted to evaluate absorption of intranasally administered creatine. Our immediate goal is to commence such an investigation using porcine nasal epithelium.

**Pharmacokinetic Analyses of Food and Non-Food-Derived Multivitamin/Mineral Formulations II: Detailed Pharmacokinetic Analysis**

Choongsung Yoo, Joungbo Ko, Dante Xing, Drew E. Gonzalez, Victoria Jenkins, Broderick Dickerson, Megan Leonard, Kay Nottingham, Jacob Kendra, Ryan Sowinski, Christopher J. Rasmussen, Richard B. Kreider*

Exercise & Sport Nutrition Lab, Texas A&M University, College Station, TX 77843, USA

*Corresponding author: rbkreider@tamu.edu

**Background**: The International Society of Sports Nutrition recommends that active individuals and athletes ingest a daily multivitamin and mineral supplement (MVM) to meet micronutrient needs. For this reason, ingestion of a daily multivitamin is a common practice to promote general health among athletes. However, there has been a debate about whether organic vitamins and minerals are more bioavailable than non-food sources of vitamins and minerals. This study compared the pharmacokinetic profiles of food-derived (FD) MVM supplements to non-food-derived (N-FD) MVM supplements.

**Materials and Methods**: In a randomized, crossover, and double-blind manner, 34 men and women fasted for 12 hours, donated a blood sample, and ingested a standardized breakfast (Nature Valley Oats ‘N Honey crunch granola bar, General Mills, Inc, Minneapolis, MN, USA) with either an N-FD MVM supplement (Nutraceutical Corp., Salt Lake City, UT, USA) or a food-derived (FD) MVM supplement (Garden of Life mykind Organics, Women’s Formula, West Palm Beach, FL, USA) supplement. Blood samples were taken 2, 4, and 6 hours after ingesting the meal and supplement. Participants observed a 7 to 14-day washout period and repeated the experiment while consuming the remaining assigned treatment. Samples were analyzed by Heartland Assays LLC (Ames, IA, USA) using standard analytic procedures. Vitamin and mineral dosage, participant weight, and serum values observed during each experiment were entered into the PK Solutions 2.0 pharmacokinetic software (Summit Research Services, Montrose, CO, USA) using single-dose analysis with 2 terms. This software calculates single-dose elimination phase and disappearance/appearance slope, rate, and half-life, as well as concentration max (Cmax), time max (Tmax), the area under the curve (AUC), the area under the moment curve (AUMC), mean residence time (MRT), volume distribution area (Vd), steady-state volume distribution area (Vss), clearance area (CL), elimination rates, and distribution/absorption rates (DA). Data were analyzed by a General Linear Model (GLM) univariate analysis with repeated measures.

**Results**: Significant differences were observed among vitamin A, E, B_12_, C, iron, and magnesium levels. There was some evidence that ingestion of an FD MVM decreased vitamin A MRT, elimination phase, and DA; vitamin E Vd, CL, and DA; vitamin B_12_ Vss and CL; vitamin C Vd, iron AUC, Vd, Vss, CL, and elimination phase half-life; and, magnesium CL while only increasing vitamin A CL observed area; vitamin C DA phase intercept; and magnesium total curve Cd (exposed) values.

**Conclusions**: Ingesting a food-derived MVN supplement does not significantly improve the volume distribution, clearance, or distribution/absorption rates of the water or fat-soluble vitamins or minerals studied compared to a non-food-derived MVM.

**Acknowledgments**: This study was funded as a grant to the Exercise & Sport Nutrition Lab at Texas A&M University by Nutraceutical/Better Being Co. (Salt Lake City, NV, USA).

**Treating the Whole Patient: Rotator Cuff Injury Secondary to Dietary Factors**

Greg E Popovich

School of Exercise Science & Athletic Training, West Virginia Wesleyan College, Buckhannon, WV, 26,201 USA

Corresponding author: popovich.g@wvwc.edu

**Background**: Protein deficiency in seniors is common, affecting greater than one-third of individuals aged ≥50 and becoming even more prevalent with advancing age. A sedentary lifestyle combined with obesity and muscle loss may lead to functional impairments that impact quality of life, loss of independence, and safety.

**Methods**: A 67-year-old female patient was referred to outpatient physical therapy for a shoulder injury. The patient’s history revealed a singular fall with no identifiable cause that resulted in a rotator cuff strain. In addition to treating the affected shoulder, the clinician desired to minimize the likelihood of re-injury from recurring falls. Therefore, a balance assessment was conducted but was unremarkable. Further examination revealed significant weakness in the lower extremities. In fact, the patient had begun living on only the first floor of her home because her stairs had become too difficult to climb. Because there was no discernable musculoskeletal or neurological cause for this weakness, a dietary analysis was performed in a separate consultation. The patient (197 lbs at 61”) revealed that in the 3 years since her retirement, she regularly consumed 5–6 cans of soda per day and had gained in excess of 30 lbs. Moreover, her weight at age 40 was 112 lbs, for a net gain of 85 lbs. Her baseline protein intake was a mere ~30 g/day. She was prescribed a daily diet of 60–80 g protein, 100–130 g carbohydrate, and 25–30 g fat (865–1,110 kcal/day) with allowance for a cheat meal every 7–10 days. Follow-up was every 1–2 weeks for 13 months. Weight and food intake were recorded daily at home.

**Results**: The client was seen for a total of 47 visits. The client demonstrated a high degree of adherence, resulting in a total loss of 41.8 lbs (21.2% reduction in body weight). There was no recurrence of falls, and–without deliberate exercise–the patient increased function to the extent that she restored her ability to negotiate stairs and regained access to the second story of her home.

**Conclusion**: Medicine as a discipline has become highly specialized, which at times leads to diagnoses being treated in a vacuum. Such focus, however, may ignore certain underlying contributing factors. This case report exemplifies just that detailing a patient who was referred to physical therapy for a shoulder injury that was ultimately traced back to lower extremity weakness caused by poor nutrition.

**Modality-Specific Differences in Measures of Performance Fatiguability Following Isokinetic Tasks**

Brady R. Fishpaw^a^*, John Paul V. Anders^a^, Balea J. Schumacher^a^, Robert W. Smith^b^, Tyler J. Neltner^b^, Jocelyn E. Arnett^b^, Terry J. Housh^b^, Richard J. Schmidt^b^, Glen O. Johnson^b^

^a^Department of Human Sciences, The Ohio State University, Columbus, OH, USA; ^b^Department of Nutrition and Health Sciences, University of Nebraska – Lincoln, Lincoln, NE, USA

*Corresponding author: fishpaw.13@buckeyemail.osu.edu

**Background**: Exercise-induced fatiguability (EF) has been quantified with maximal voluntary isometric (IM) and maximal isokinetic (IK) muscle actions before and after a fatiguing task. It remains unclear if EF exhibits modality-specific differences based on the type of fatiguing tasks performed. The purpose of the present study was to determine whether IM and IK muscle actions exhibit differences in peak force (PF), electromyographic amplitude (EMG AMP), and EMG mean power frequency (MPF) following IK fatiguing tasks.

**Methods**: Eleven recreationally trained men (age: 20.8 ± 1.7 years; body mass: 84.0 ± 16.2 kg; height: 179.3 ± 7.2 cm) performed unilateral (UL), 6-second IMs and UL, maximal IK leg extensions at 180°·s^−1^ before and after fatiguing tasks consisting of either 50 bilateral (BL) or 50 UL maximal, IK leg extensions at 180°·s^−1^ in random order on separate days. PF and EMG AMP and EMG MPF of the left vastus lateralis were examined by separate 2(Modality: IK, IM) x 2(Fatigue: UL, BL) x2 (Time: Pre-fatigue, Post-fatigue) repeated measures ANOVAs. Follow-up Bonferroni-corrected pairwise comparisons were conducted when appropriate. An alpha value of *p* < 0.05 was considered statistically significant.

**Results**: For PF, there was no significant 3-way interaction, but a significant Modality x Time interaction (*p* = 0.002, η^2^_p_ =0.64). The post-hoc analysis for the IK modality demonstrated significantly (*p* < 0.001, *d* =1.37) greater pre- (53.93 ± 9.77 kg) than post-fatigue (41.54 ± 8.18 kg) force values, while the IM modality demonstrated no significant differences (*p* >0.05) across Time. For EMG AMP, there were no significant 3- or 2-way interactions, but a main effect for Modality (*p* < 0.001, η^2^_p_ =0.915). The *post-hoc* analysis for modality demonstrated that the IK EMG AMP (661.97 ± 232.55 µV) was significantly (*p* < 0.001, *d* =1.25) greater than IM EMG AMP (406.00 ± 173.58 µV). For EMG MPF, there were no significant 3- or 2-interactions, but significant main effects for Time (*p* = 0.002, *d* =0.62) and Modality (*p* = 0.001, *d* =0.36). The *post-hoc* analysis for Modality demonstrated that the IM EMG MPF (75.26 ± 14.82 Hz) was significantly greater (*p* = 0.045, *d* =0.36) than IK EMG MPF (70.45 ± 12.07 Hz). The *post-hoc* analysis for Time demonstrated that pre- (77.03 ± 14.87 Hz) was significantly (*p* = 0.002, *d* =0.62) greater than post-fatigue (68.68 ± 11.94 Hz).

**Conclusions**: Only the IK Modality exhibited EF following the IK fatiguing tasks. Both modalities exhibited similar declines in EMG MPF, which suggested the development of peripheral fatigue. The IK modality exhibited a greater EMG AMP than the IM Modality regardless of EF. These results suggested that EF should be assessed with tasks that match the fatiguing modality. A modality-matched task may be more effective at assessing the exercise-induced changes in maximal force and neuromuscular excitation.

**Does a Caffeinated Fitness Drink Affect Performance and Physiological Responses during a VO_2_max Test? – A Pilot Study**

Rachel Chenoweth and Zidong Li

Department of Molecular Biology and Chemistry, Christopher Newport University, Newport News, VA, USA

Corresponding author: rachel.chenoweth.19@cnu.edu

**Background**: Caffeinated sports performance drinks are marketed for their ergogenic benefits when consumed before exercise. Many sources of caffeine in energy drinks are sourced in part from guarana extract, which is deemed illegal under the National Collegiate Athletic Association (NCAA) Banned Substance Policy. The present pilot study was conducted to examine if consuming these drinks may have an acute effect on performance (i.e. maximal oxygen consumption, or VO_2_max), perceived exertion at the first ventilatory threshold (VT1), as well as maximal heart rate (HRmax), maximal respiratory exchange ratio (RER), and time to exhaustion (TTE) during a graded exercise test (GXT) on treadmill.

**Methods**: Using a randomized, counterbalanced design, six healthy college students (5 female, 1 male; age 21.3 ± 0.5 years, body mass 67.9 ± 11.7 kg) completed two conditions: 1) a control GXT (CON) and 2) a GXT 15 minutes after consumption of a 355 ml strawberry guava flavored caffeinated fitness drink (Celsius®) containing 200 mg caffeine (CAF). Exercise protocols were customized to each subject’s fitness level to induce fatigue within 8 to 12 minutes. Workload was increased and ratings of perceived exertion (RPE) on Borg’s 6-20 scale were obtained every minute. HR was measured using a Polar H10 sensor. Subjects’ expired gas was analyzed using a ParvoMedics TrueOne 2400 metabolic system and further processed using the ‘11-breath rolling average’ method. The ‘V-slope’ method was used to determine the VT1. Mean values between conditions were compared using paired sample t-tests in Microsoft Excel (significance level *p* < 0.05).

**Results**: Four out of the six subjects had a slightly higher VO_2_max in CAF as compared to CON. However, it was not significantly different when comparing the mean values (CAF: 44.2 ± 8.5 ml/kg/min; CON: 43.4 ± 7.7 ml/kg/min, *p* = 0.48). Similarly, there was no significant difference in HRmax (CAF: 199 ± 6 beats/min; CON: 200 ± 6 beats/min, *p* = 0.23), RPE at VT1 (CAF: 8 ± 1; CON: 8 ± 1, *p* = 0.70), maximal RER (CAF: 1.11 ± 0.05; CON: 1.12 ± 0.05, *p* = 0.71) or TTE (CAF: 8.59 ± 2.10 min; CON: 9.10 ± 2.34 min, *p* = 0.35) between the conditions.

**Conclusions**: The preliminary findings suggest that consuming a single dose of a caffeinated fitness drink did not significantly enhance performance or alter physiological responses when running with an incremental workload toward exhaustion. Further studies with greater sample sizes are warranted.

**The Differential Effects of EPA, DHA, or EPA + DHA Supplementation on Whole Blood Polyunsaturated Fatty Acids**

Jeffery L. Heileson^a,b^*, Robert B. Wallace^b,c^, Aireal S. Williams^b^, Jane E. Carnett^b^, William C. Hall^b^, Carla J. Trochez^b^, Jeffrey S. Forsse^a^, LesLee K. Funderburk^a^

^a^Baylor University, Waco, TX, USA; ^a^Walter Reed National Military Medical Center, Bethesda, MD, USA; ^c^William Beaumont Army Medical Center, Fort Bliss, TX, USA

*Corresponding author: jeffery_heileson@baylor.edu

**Background**: Globally, long-chain omega-3 fatty acid dietary intake is suboptimal as evidenced by low omega-3 fatty acid status (omega-3 index [O3i], % eicosapentaenoic acid [EPA] + % docosahexaenoic acid [DHA] in red blood cells). High-dose fish oil supplementation (> 2 g∙d^−1^) is an established strategy to significantly increase whole blood fatty acid status within two weeks. However, the fatty acid status response to supplementation may vary based on the type of omega-3 fatty acid. This study aimed to determine the differential effects of EPA, DHA, or EPA + DHA supplementation on whole blood omega-3 fatty acids.

**Methods**: Twenty-three men were randomized to 4 g∙d^−1^ EPA (*n* = 8), DHA (*n* = 7), or EPA + DHA (*n* = 8) supplementation for seven weeks. Participants provided dried blood spots via fingerstick before (PRE) and after (POST) supplementation. Whole blood polyunsaturated fatty acid status (EPA, DHA, docosapentaenoic acid omega-3 [DPA], alpha-linolenic acid [ALA], linoleic acid [LA], arachidonic acid [AA]) was analyzed by a third-party laboratory (OmegaQuant LLC, Sioux Falls, SD, USA). A mixed-model repeated measures ANOVA (Group: 3 levels, Time: 2 levels) was used for analysis.

**Results**: The O3i and EPA increased across all groups (*p* < .001). DHA increased in the EPA + DHA (*p* < .001) and DHA (*p* < .001) groups; DHA remained unchanged in the EPA group (*p* = .640). DPA increased following supplementation of EPA (*p* < .001) and EPA + DHA (*p* = .022). AA decreased in the EPA (*p* = .022) and DHA (*p* = .005) groups. At POST, EPA status was higher in the EPA group compared to DHA (*p* = .001), whereas DHA status was higher in the EPA + DHA (p < .001) and DHA (*p* < .001) compared to the EPA group. The DPA status was higher in the EPA group compared to EPA + DHA (*p* < .001) and DHA (*p* < .001). No group-by-time interactions were observed for O3i, ALA, AA, and LA.

**Conclusions**: High-dose long-chain omega-3 supplementation increased O3i and EPA within seven weeks regardless of omega-3 type (EPA, DHA, or both), although the magnitude varied. Differential effects were observed for most other whole blood polyunsaturated fatty acids, especially the response of whole blood DHA and DPA. Specific omega-3 fatty acids can be consumed to produce a more targeted intervention based on the goal of preconditioning, e.g. higher DHA content in those exposed to head impacts (football, rugby).

**Efficacy and Safety of 8-weeks Supplementation of a Fenugreek Extract on Endurance Capacity in Healthy Young Males: A Double-Blind, Placebo-Controlled Study**

Dean Mills^a^, David Briskey^b^, Michael Sedlak^c^*, Dhananjay Raje^d^, Pallavi Deshpande^e^, Prasad Thakurdesai^e^

^a^The University of Southern Queensland, Cardiorespiratory Clinic, Ipswich Campus, 11 Salisbury Rd, Ipswich, QLD, 4305, Australia; ^b^RDC Clinical, Unit 6/76 Doggett Street, Newstead, Brisbane, QLD, 4006, Australia; ^c^CPC Nutrition, London, Ontario, Canada; ^d^Alfa Level Consultants, 96, East Samarth Nagar, Wardha Road, Nagpur-440,015, India; ^e^Indus Biotech Limited, 1, Rahul Residency, Off Salunke Vihar Road, Kondhwa, Pune-411,048, India

*Corresponding author: Michael@natrusolate.com

**Background**: Endurance capacity is an important component to support sustained physical activity. This study assessed the efficacy and safety of fenugreek extract supplementation on endurance capacity in exercising, healthy young males.

**Methods**: Capsules containing 600 mg of a standardized extract from fenugreek seeds (with select glycosides, 4‑hydroxyisoleucine and trigonelline) (Enducor®) or matching placebo was orally supplemented to 100 exercising young males (18-40 years), with healthy BMI for 8 weeks. The subjects were on aerobic exercise training sessions 4 times a week. The primary efficacy outcome was time to exhaustion using treadmill testing. Secondary efficacy outcomes included parameters of cardio-respiratory [total distance run, maximal oxygen consumption (VO_2Max_), maximal heart rate (HR_Max_), systolic and diastolic blood pressure, respiratory exchange ratio, HR at gas exchange thresholds 1 and 2 (GET1 and GET2), velocity at VO_2_ (vVO_2Max_) and VO_2Max_ while on treadmill], energy expenditure [metabolic equivalent (MET), fasting plasma glucose, plasma non-esterified fatty acids (NEFA)], whole-body fatigue [Multidimensional Fatigue Symptom Inventory-Short Form (MFSI-SF)], quality of life by 36-item Short Form (SF-36) survey. Safety parameters were analyzed included any adverse events, serum alanine transaminase, aspartate transaminase, creatinine, sodium, and potassium. All the parameters were assessed at the baseline, week 4 and week 8, except blood parameters which were assessed at baseline and week 8. The data analysis was performed using SPSS software (version 26.0), with p < 0.05 considered significant.

**Results**: Fenugreek extract supplementation showed a statistically significant increase in time to exhaustion, total distance run, VO_2Max_, vVO_2Max_ and MET (p < 0.01), VO_2_ at GET1 and velocity at GET2) (p < 0.05) with significant decreases in NEFA (p < 0.01), HR_Max_ and mental fatigue domain of MFSI-SF (p < 0.05), as compared with baseline values. There were no significant differences in any parameters from baseline to week 4 or week 8 in the placebo supplemented group. The values of the safety parameters did not show significant differences in either group during the 8 week supplementation period. No adverse events were reported.

**Conclusions**: Fenugreek extract supplementation significantly improved time to exhaustion, cardio-respiratory parameters, energy expenditure, and mental fatigue. It was found to be safe and well tolerated.

**Acknowledgments**: Financial support by Indus Biotech Limited, Pune, India.

**Cardiovascular and Metabolic Cost During Upper and Lower Body High-Load Resistance Training Sessions in Trained Men and Women: A Pilot Study**

Matías Monsalves-Álvarez^a^*, José Gomez-Lopez^b^, Francisco Sanchez^b^, Sofia Bobadilla^b^, Sebastián Jannas-Vela^a^, Denisse Valladares^a^, Claudio Pérez de Tudela^b^ and Nicole Jeria^b^

^a^Instituto de Ciencias de la Salud, Universidad de O´Higgins, Rancagua, Chile

^b^Motion Health and Performance Center, Lo Barnechea, Chile

*Corresponding author: matias.monsalves@uoh.cl

**Background**: Resistance training (RT) is the primary exercise modality used for improving body composition, health, and performance. Proposed mechanisms for increasing muscle size and strength rely on the local metabolic ‘stress’ induced by weight and repetitions during RT. Metabolically, RT relies primarily on the use of the phosphagen system, glycogenolysis, and intracellular lipid utilization, which have been evaluated with muscle biopsies. However, this metabolic characterization during RT exercise sessions has primarily focused on lower body exercises with a bodybuilding approach that does not reflect programs applied to body composition, which consider full-body routines or circuits. Therefore, this study aims to analyze cardiovascular and metabolic variables in upper and lower-body high-load resistance training sessions in trained men and women.

**Methods**: Five men and woman (2/3, respectively) with at least 3 years of experience in RT underwent baseline evaluation of; one-repetition maximum, resting energy expenditure by indirect calorimetry (RMR, Q-NRG, Cosmed), maximal oxygen consumption, and creatine kinase (CK) determination. Subjects performed two different high-load training sessions upper (UP) and lower (LO) at 75% RM on nonconsecutive days wearing a portable spiroergometer (Pnoe®, Greece) during the whole training. UP session included the following: Bench and shoulder press, pulldown, unilateral dumbbell row, stating bicep curl and triceps pulldown, while the LO session: Smith back squat, split squat, RDL, hip thrust, leg extension, and leg curl. One day before and after training sessions, RMR was determined while CK was evaluated before, immediately and 24 h after each session.

**Results**: All subjects performed three sets of 12 repetitions with 1-2 repetitions in reserve (RIR). VO_2_ (LO,18.08 ± 2.4; UP,13.17 ± c.2 ml·kg·min^−1^; p =0.001), VE (LO,74.8 ± 12.8; UP, 50.7 ± 6.7 L/min: p =0.002) and HR (LO,126.4 ± 7.2; UP, 109.9 ± 9.5 bpm, p =0.007) where significantly higher on LO compared to UP sessions. Concerning energy cost, LO training sessions consider a higher absolute (LO,6.4 ± 1.0; UP, 4.7 ± 0.45 kcal/min) and relative (LO, 0.08 ± 0.01; UP, 0.06 ± 0.00 kcal/body weight) metabolic cost. LO session also elicits a higher CHO_ox_ compared with LO; 1.8 ± 0.65 and 1.04 ± 0.2 g/min (p =0.02), respectively. Also, resting metabolic rate (RMR) and VO2 decrease 24 h after in UP (p =0.04) while no changes were observed post LO training sessions. Interestingly, CK levels showed no changes immediately after training, but were significantly higher at baseline and after 24 h LO sessions (p =0.04 and 0.03), respectively.

**Conclusions**: We conclude than upper and lower high-load resistance training sessions possess important cardiovascular and energetic cost differences that needs to be considered when performed, especially regarding resting and nutritional aspects. The present data will help dietitians and trainers to order in a more precise manner the RT session based on metabolic variables.

**Acknowledgments**: Jose Gomez-Lopez is the CEO of Motion Health and Performance Center where training and evaluations were carried out.

**Development of a Sustained Release Caffeine: Optimizing Encapsulation Level for Slow Release**

Lindsey Ormond^a^*, Olivia Drehmel^a^, Peter Yoder^a^, Robert Wildman^b^

^a^Milk Specialties Global, Eden Prairie, MN, USA; ^b^Kansas State University, Manhattan KS, USA

*Corresponding author: lormond@milkspecialties.com

**Background**: Caffeine is recognized as the most widely available and commonly consumed stimulant globally. It is found naturally in many foods and beverages, as well as being a stable of pre-workout and energy products, improving both physical and mental performance. Doses of 3–6 mg/kg BW can improve physical performance, although improvements may be seen as low as 2 mg/kg BW. However, higher caffeine doses are associated with negative side effects, such heart palpitations, anxiety, headaches, and hindered sleep quality. Sustained caffeine release over several hours may be a strategy to provide the benefits of caffeine over an extended period while avoiding these negative side effects. Sustained release caffeine has previously been shown to significantly improve upper body reaction time compared to placebo during hours 5-8 after consumption; however, there are few ingredients in the marketplace that provide sustained release. Therefore, the purpose of this study was to assess the rate of caffeine released for a new encapsulated caffeine ingredient, comparing three different levels of encapsulation, to unencapsulated caffeine.

**Methods**: Three levels of caffeine encapsulation – low, medium, and high – were made utilizing a well-tested encapsulation substrate. Standard anhydrous caffeine was used as the unencapsulated control. Dissolution was used to assess the percent of caffeine released over 8 hours, with measurements taken at 30 mins, 45 mins, 1 hr, 2 hr, 4 hr, 6 hr and 8 hrs. The dissolution method employed hydrochloric acid (HCl) to reduce the pH to pH 2.0 for 2 hrs, before being increased to pH 6.8 with trisodium phosphate (TSP). The solution was maintained at a temperature 37°C ± 0.5. R was used to complete statistical analysis.

**Results**: Compared to control, all levels of encapsulation significantly prolonged caffeine release (*P* < 0.001). Slower release of caffeine was seen with increasing levels of encapsulation (30 min – low coating: 74.0 ± 1.67%; medium coating: 50.7 ± 2.05%; high coating: 30.7 ± 1.67%), meanwhile unencapsulated caffeine was almost completely released by 30 minutes (98.3 ± 1.67%). Low encapsulation level peaked at 2 hours (97.1 ± 1.67%), with medium and high encapsulation still releasing through 8 hours (medium coating: 90.6 ± 2.05%; high coating: 83.7 ± 1.67% at 8hrs).

**Conclusions**: Increasing levels of encapsulation of caffeine led to significantly slower caffeine release over 8 hr in dissolution, while unencapsulated caffeine was completely released within 30 minutes. These data highlight that encapsulation of caffeine enables sustained release over a period of 4-8 hours, which could be beneficial for prolonged or delayed performance. Future research should focus on the performance implications of this sustained release.

**Acknowledgments**: This study was funded by Milk Specialties Global

**Figure:**
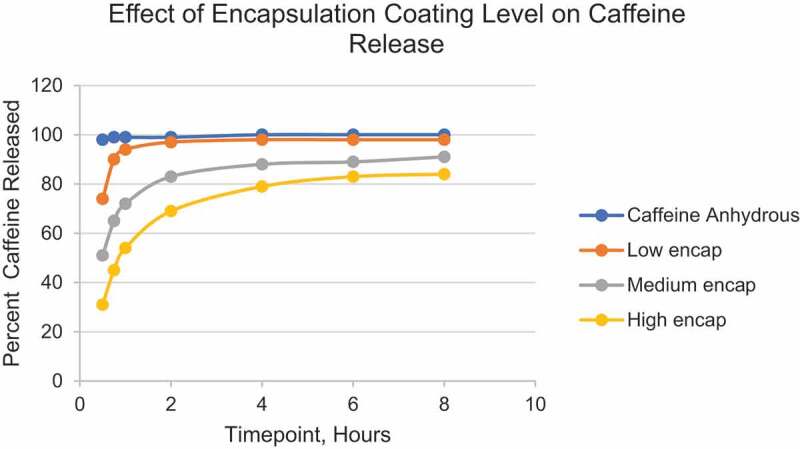

**Changes in Deoxy[heme] Levels and Metabolic Efficiency During Repeated Maximal Intermittent Handgrip Exercise**

Modesto A. Lebron, Justine M. Renziehausen, Ethan C. Hilla, Jeffrey R. Stout, David H. Fukuda

School of Kinesiology and Rehabilitation Sciences, University of Central Florida, Orlando, FL

**Background**: The maximal handgrip strength assessment is often used to indicate overall muscular strength and functional disabilities. However, many daily activities, sports, and occupations require repeated or sustained maximal handgrip effort. Near-infrared spectroscopy (NIRS) used to examine tissue oxygenation, may allow for an indirect estimate of metabolic efficiency by comparing the ratio of force output to blood flow in the active musculature. The purpose of this study was to observe the relationship between hand grip force and muscle hemodynamics during an intermittent handgrip fatiguing protocol.

**Methods**: Eight males and seven females (mean± SD; 24.4 ± 6.5 years, 170.4 ± 7.28 cm, 75.2 ± 13.5 kg) completed one familiarization trial and two identical experimental trials of an intermittent handgrip fatiguing protocol separated by a minimum of 24 hours. The protocol consisted of 60 consecutive 4-second maximal effort isometric contractions with a 1-second rest period between each contraction. Force data were recorded continuously with a dynamometer, and the peak force was identified for each repetition. Simultaneously, a NIRS device measuring changes in oxygenated and deoxygenated hemoglobin (deoxy[heme]) was placed on the surface of the flexor digitorum superficialis muscle and secured with colored athletic tape to prevent light contamination. Changes in deoxy[heme] were measured for contractions 1, 15, 30, 45, and 60 as a percentage of the baseline (%HHb). The baseline measure was identified as the average value over the first two minutes of NIRS measurements obtained at rest before the protocol began. Metabolic efficiency (ME) for each contraction was calculated as peak force/%HHb. Differences in %HHb and ME were assessed with separate two-way (repetition x trial) repeated measures ANOVAs.

**Results**: No repetition x trial interactions were observed for %HHb (F =0.624, *p* = 0.583, η_p_^2^ =0.043) or ME (F =2.29, *p* = 0.146, η_p_^2^ =0.141). While no main effects of trial were observed (*p* >0.05), main effects of repetition were found for %HHb (F =18.017, *p* < 0.001, η_p_^2^ =0.563), and ME (F =18.017, *p* < 0.001, η_p_^2^ =0.670). Post hoc analysis revealed that %HHb during repetition 1 (81.3 ± 16.5%) was significantly lower (*p* < 0.001) than repetitions 15, 30, 45, and 60 (118.1 ± 25.7% to 121.7 ± 33.8%). Conversely, ME for repetition 1 (45.1 ± 25.3 kgf/%HHb) was significantly higher (*p* < 0.001) than repetitions 15, 30, 45, and 60 (12.0 ± 4.05 to 18.1 ± 5.98 kgf/%HHb).

**Conclusion**: This study observed an increase in deoxy[heme] and a decrease in ME between the 1^st^ and 15^th^ repetitions of the maximal intermittent handgrip protocol, with no differences in the responses between assessment trials. This rapid hemodynamic response warrants further precise investigation. The consistency of this response throughout the protocol could be valuable in evaluating ergogenic aids intended to enhance blood flow during exercise.

**Is the Resting Metabolic Rate Ratio a Good Proxy Indicator of Energy Deficiency in Men? A Preliminary Study**

Ana Carla Chierighini Salamunes, Nancy I.Williams, Mary Jane De Souza

Pennsylvania State University, University Park, PA, USA

Corresponding author: mjd34@psu.edu

**Background**: Active individuals who have low energy availability may experience an energy deficiency, as evidenced by low serum total triiodothyronine (TT3). The ratio of measured-to-predicted resting metabolic rate (RMRratio) has been established as a proxy indicator of energy deficiency and metabolic compensation in active women, but parameters for men have not yet been established. The purpose of this preliminary study was to determine if the RMRratio is associated with TT3 in active young men.

**Methods**: Subjects were men aged 22 ± 0.6 years (n =36), with a body mass index of 16-29.9 kg/m^2^, who purposefully exercised for a minimum of 150 min/week and achieved a maximal oxygen consumption of at least 44 mlO_2_/kg/min on a graded treadmill maximal test. Dual-Energy X-Ray Absorptiometry (DXA) assessed body composition. Subjects reported to the laboratory in the morning after a 12-h fast and abstaining from physical activity. RMR was assessed via indirect calorimetry, and a blood draw was obtained to assess serum TT3. The equations of Cunningham_1980_, Cunningham_1991_, Harris-Benedict, and Hayes (DXA-predicted) were utilized to calculate the predicted RMR. The ratio of the measured RMR to the predicted RMR was calculated for each of the four equations. Pearson’s correlation coefficients and simple linear regressions were fitted to determine the relationship between the four RMR ratios and TT3. Data are presented as mean ± SEM.

**Results**: The DXA-predicted RMRratio (0.957 ± 0.015) was positively correlated with TT3 (111.9 ± 3.66 ng/dl) (r =0.406, R^2^ =0.165, p =0.014), and the Harris-Benedict RMRratio (0.952 ± 0.019) was negatively correlated with TT3 (r =−0.569, R^2^ =0.324, p < 0.001). The Cunningham_1980_ (0.959 ± 0.014) and Cunningham_1991_ RMRratios (1.010 ± 0.014) were not significantly correlated with TT3 (respectively, r =0.248, R^2^ =0.061, p =0.145, and r =0.286, R^2^ =0.082, p =0.091). The DXA-predicted RMRratio was strongly correlated with the Cunningham_1980_ (r =0.944, R^2^ =0.892, p < 0.001) and the Cunningham_1991_ RMRratios (r =0.952, R^2^ =0.907, p < 0.001), but not with Harris-Benedict (r =0.233, R^2^ =0.054, p =0.172).

**Conclusions**: The Harris-Benedict RMR ratio does not seem useful to predict TT3, likely because the equation does not account for differences in body composition. Cunningham RMR ratios may be useful indicators of energy deficiency in exercising men, but further investigation is needed to determine a possible relationship. The DXA-predicted RMR ratio has the potential to be used as a proxy indicator of energy deficiency in men, but more data is necessary to confirm that assumption. At this time, it is premature to determine a cutoff threshold of RMRratio to be used as an indicator of energy deficiency in exercising men.

**Acknowledgments**: Ana Carla Chierighini Salamunes is funded by the Fulbright Commission Brasil and CAPES.

**Iron Deficiencies Following Pre-Season Training in NCAA Division I Acrobatics & Tumbling Student-Athletes**

Leticia C. de Souza^a^*, Tomas J. Chapman-Lopez^b^, Katherine M. Lee^a^, Jeffery L. Heileson^a,c^, Ricardo Torres^b^, Dillon R. Harris^a^, Kathleen A. Richardson^a^, Jeffrey S. Forsse^b^,Andrew R. Gallucci^a^, LesLee K. Funderburk^a,d^*

^a^Department of Health, Human Performance, and Recreation, Baylor University, Waco, TX 76706, USA; ^b^Integrated Laboratory of Exercise, Nutrition, and Renal Vascular Research, Baylor University, Waco, TX 76706, USA; ^c^Nutrition Services Division, Walter Reed National Military Medical Center, Bethesda, MD 20889, USA; ^d^Human Sciences and Design, Baylor University, Waco, TX 76706, USA

Corresponding Author: Leti_de_Souza@baylor.edu

**Background**: Iron plays an essential role in oxygen transport and energy metabolism. Despite its importance, female athletes are susceptible to iron deficiency due to several reasons, including inadequate dietary intake, excessive exercise, and heavy menstrual bleeding. Non-anemic iron deficiency (low serum ferritin [SF] in the condition of normal hemoglobin levels) can lead to fatigue and weakness, wherein it may negatively impact athletic performance. This study aimed to investigate the iron-deficiency related biomarkers of A&T student-athletes and their respective changes following pre-season training.

**Methods**: Thirty-seven female NCAA Division I A&T student-athletes were examined (age: 19.55 ± 1.11 years; height: 159.77 ± 5.85 cm, weight: 61.76 ± 9.82 kg; fat-free mass: 46.32 ± 6.27 kg; body fat percentage: 24.51 ± 4.84%) at the beginning (October [T1]) and cessation of their pre-season training (December [T2]). All student-athletes reported to the laboratory on two occasions, providing plasma and serum samples to assess iron-deficient related biomarkers (i.e. SF, hemoglobin, red blood cell distribution width) and female hormones (i.e. estradiol, progesterone). A paired sample t-test assessed significant differences before and after preseason training.

**Results**: Analyses revealed significant declines in SF (T1: 43.9 ± 22.03; T2: 26.1 ± 12.51 ng/mL, *p* < .001) and total protein (T1: 7.24 ± 0.38; T2: 6.90 ± 0.43 g/dL, *p* < .001) following two months of pre-season training. At T1, 8% (*n* = 3) of student-athletes presented SF < 20 ng/mL (suggested stage 1 iron-deficient erythropoiesis), while 46% (*n* = 17) had SF < 35 ng/mL (suggested stage 1 iron-depletion). Contrariwise, at T2, 43% (*n* = 16) had SF < 20 ng/mL and 73% (*n* = 27) of student-athletes displayed SF< 35 ng/mL. No significant relationships were found in hemoglobin, estradiol, and progesterone concentrations between timepoints.

**Conclusion**: These findings support A&T student-athletes experience significant declines in SF during pre-season training. Most student-athletes presented low SF concentrations (< 35 ng/mL) at T2, which is considered the threshold for required treatment in athletes. Consequently, A&T student-athletes would benefit from careful monitoring and nutrition counseling on adequate iron intake, especially following intensive training periods to ensure normative SF ranges are met. Future studies should evaluate female athletes from different sports to ensure maintenance of adequate SF concentrations to decrease the risk of iron deficiency.

**The Influence of CYP1A2 Genotypes on the Impact of Caffeine Ingestion on Subjective Outcomes in Females**

Jessica M. Prather^a^*, Christine M. Florez^a^, Amie Vargas^a^, Bella Soto^a^, Audrey Ross^a^, Abby Harrison^a^, Ariane Secrest^b^, Darryn Willoughby^a,c^, Sydney Kutter^a,c^, Lem W. Taylor^a,d^

^a^Human Performance Lab, University of Mary Hardin-Baylor, Belton, TX, USA; ^b^Public Health Program, University of Mary Hardin-Baylor, Belton, TX, USA; ^c^Physician Assistant Program, University of Mary Hardin-Baylor, Belton, TX, USA; ^d^Doctor of Physical Therapy Program, University of Mary Hardin-Baylor, Belton, TX, USA

Corresponding author: jprather@mail.umhb.edu

**Background**: Caffeine is a neurostimulator that binds to adenosine receptors, allowing individuals to decrease perceived fatigue and increase excitability. Caffeine is metabolized in the liver primarily by the cytochrome P450 1A2 (CYP1A2) enzyme and has two genotypes: slow (AC/CC) vs. fast (AA) metabolizer. Thus, this experiment investigated the impact that CYP1A2 genotypes have on the cognitive effects of caffeine in females.

**Methods**: In this double-blind, placebo-controlled, crossover study, 36 caffeine-habituated females (AA, n =20, 21.6 ± 2.2 y, 163.2 ± 9.6 cm, 70.3 ± 15.4 kg; AC/CC, n =16, 22.3 ± 3.8 y, 165.6 ± 5.3 cm, 68.7 ± 14.3 kg) provided baseline assessments for subjective outcomes including energy, focus, concentration, alertness, and fatigue. The total score was calculated by summing each participant’s subjective outcome ratings. Participants ingested their randomly assigned treatment (placebo-PL or 6 mg/kg-bw anhydrous caffeine-CAF) and repeated the assessment at 30-, 60-, and 120-minutes post-ingestion and following a standardized bout of resistance exercise and repeated the protocol on a subsequent day. Adverse effects were assessed at the end of each testing session. CYP1A2 genotype was determined by 23 and Me using saliva. Data were analyzed using a two-way ANOVA with repeated measures. The follow-up assessment data were analyzed using a paired samples t-test with Cohen’s d effect sizes. Significance was accepted *a priori* at p < 0.05.

**Results**: Results of the two-way ANOVA revealed a significant treatment x genotype interaction for the subjective outcome index score (p = 0.045) and a significant difference for time (p < 0.01) and between genotype (p < 0.001) were uncovered. A follow-up analysis revealed a higher total score (p = 0.028) in CAF vs. PL in the AA group and a lower total score (p < 0.01) in the CAF vs. PL in the AC/CC group. The adverse effects data revealed a significant difference between treatments for dizziness (p = 0.014; ES 0.725) in the slow (AC/CC) genotype group and the dizziness data for the fast (AA) genotype group was approaching significance (p = 0.056; ES 0.468).

**Conclusions**: These findings are the first to study the genotype influence on subjective outcomes in a female population. The CYP1A2 AA and AC/CC groups experienced similar responses for the individual subjective outcomes and most follow-up adverse effects, but caffeine ingestion in the fast genotype group did improve the subjective outcomes total score with no observed benefit in the slow group. The observed response for dizziness should be considered and may be attributed to the high dose used here.

**Acknowledgments**: This study was funded by a UMHB Graduate Faculty Research Grant. The authors declare no conflicts of interest.

**Efficacy of a Microalgae Extract from *Porphyridium Cruentum* in Regulating Muscle Recovery Parameters Following Exhausting Exercise in Mice**

Jonathan Maury^a^*, Sicard Flavie^b,c^, Jean-François Landrier^b,c^, David Fukuda^d^,Jeffrey Stout^d^, Rémi Pradelles^a^

^a^Microphyt, Research & Development Department, 34,670 Baillargues, France; ^b^Aix-Marseille Université, C2VN, INRAE, INSERM, 13,005 Marseille, France; ^c^PhenoMARS Aix-Marseille Technology Platform, CriBiom, 13,005 Marseille, France; ^d^Institute of Exercise Physiology and Rehabilitation Science, School of Kinesiology and Physical Therapy, University of Central Florida, Orlando, FL, USA.

*Corresponding author: jonathan.maury@microphyt.eu

**Background**: A unicellular species within red algae family, *Porphyridium cruentum* may have benefits in sport nutrition field due to its content of several molecules of interest including phycoerythrin, exopolysaccharides, and amino acids. Beyond the anti-fatigue role described in the literature, the combination of these different molecules may optimize muscle recovery following exhausting exercise by improving fuel storage and muscle repairment through different inflammatory response and oxidative stress-related signaling pathways. Thus, the purpose of this *in vivo* study was to evaluate the efficacy of a microalgae extract from *Porphyridium Cruentum* on muscle recovery parameters in mice.

**Methods**: A total of 72 mice were used to conduct this experimental study, equally divided into four groups. Beyond the control group, three doses of microalgae extract were evaluated, 50 mg/day (VLD); 100 mg/day (LD) and 500 mg/day (HD), in human equivalent. After 4 weeks of daily supplementation, the mice were subjected to a downhill running protocol for 120 minutes (−16° at 20 meters per minute). Plasma sample, liver, and muscle were collected 24 or 48 hours after exercise to evaluate several parameters related to muscle damage, inflammation, lipid and carbohydrate metabolisms, and muscle gene expression of Mcp-1, Foxo-1, F4/80, Vegf and Nrf2 targeted genes.

**Results**: No statistically significant discrepancy were described between groups regarding body weight gain, food intake, and the running distance during exercise protocol. Compared to the control group, blood levels were significantly reduced for IL-6 (p < 0.05), IL-1α (p < 0.05), IL-10 (p < 0.01) at 48 h and IL-17 (p < 0.01) at 24 h in LD and HD groups. Liver glycogen level is significantly higher in the LD and HD groups compared to control (p < 0.05) 24 h after exercise (without statistically significant significance between LD and HD groups in muscles). Regarding muscle gene expressions, mRNAs levels of TNFa, Il-6 myokines, and macrophage marker F4/80 were significantly induced in LD and HD groups compared to control at 24 h (p < 0.01). Muscle Gclc and Gclm mRNAs (Nrf2 pathway markers) levels were induced in the HD group compared to the control group at 24 h (p < 0.01).

**Conclusions**: Results provide some evidence that supplementation with a microalgae extract from *Porphyridium Cruentum*, particularly in LD and HD groups, regulates physiological response leading to optimized muscle recovery after an exhausting exercise. Beyond the improvement of Cori cycle allowing to a rapid restoration of liver glycogen after exercise, the supplementation seems to enhance inflammatory and oxidative responses leading to a muscle repairment phenotype characterized notably by the recruitment and/or differentiation of macrophages.

**Acknowledgments**: This study was funded by MicroPhyt (Baillargues, FR) as a fee-for-service project to the PhenoMARS Aix-Marseille Technology Platform, CriBiom (Marseille, FR). Data were collected and analyzed by SF and JFL from PhenoMARS/Cribiom platform.

**Effects of Liposomal Ashwagandha Supplementation on Markers of Health and Safety in Healthy Adults**

Dante Xing^a^, Megan Leonard^a^, Landry Estes^a^, Broderick Dickerson^a^, Drew E. Gonzalez^a^, Sarah Johnson^a^, Victoria Jenkins^a^, Joungbo Ko^a^, Choongsung Yoo^a^, Martin Purpura^b^, Ralf Jäger^b^, Ryan Sowinski^a^, Christopher J. Rasmussen^a^, Richard B. Kreider^a^*

^a^Exercise & Sport Nutrition Lab, Texas A&M University, College Station, TX 77843, USA; ^b^Increnovo LLC, Whitefish Bay, WI 53217, USA

*Corresponding Author: rbkreider@tamu.edu

**Background**: Ashwagandha has been used in traditional Ayurvedic medicine as an adaptogen to reduce stress, improve memory and cognitive function, and improve exercise performance. We recently reported that acute ingestion of 400 mg of ashwagandha improved selected measures of executive function, helped sustain attention, and increased short-term/working memory. The purpose of this study is to determine the safety of 30 days of ashwagandha supplementation in healthy adults.

**Methods**: 59 men and women (22.7 ± 7 yrs, 74.9.1 ± 16 kg, 26.2 ± 5 BMI) fasted for 12 hours, donated a blood sample and completed a comprehensive side effect survey. In a randomized and double-blind manner, participants were then administered 225 mg of a placebo (gum Arabic, PLA) or a liposomal ashwagandha (*Withania somnifera*) root and leaf extract (NooGandha®, Specnova LLC, LLC, Tysons Corner, VA, USA, ASH) for 30-days. Participants then returned to the lab to repeat the experiment. Whole blood was assayed for cell blood counts (CBC) with percent differentials, while serum samples were assayed for standard clinical chemistry markers. Data were analyzed by a General Linear Model (GLM) univariate analyses with repeated measures and mean changes from baseline with 95% Confidence Intervals (CI). Chi-square analysis was used to evaluate responses to the side effects.

**Results**: Multivariate analysis revealed no significant treatment x time effects in CBC (*p* = 0.291, $\eta_{p}^{2}$ = 0.281, large effect) or serum blood markers (*p* = 0.102, $\eta_{p}^{2}$ = 0.530, large effect). Pairwise analysis revealed that post-supplementation (D30) lymphocyte levels were significantly higher after 30-days (D30) with PLA (4.98 % [0.5, 9.5], *p* = 0.03) while calcium (0.016 mg/dL [−0.03, 0.35], *p* = 0.097) and total protein (0.16 mg/dL [−0.03, 0.36], *p* = 0.096) tended to be higher although values were well-within clinical norms. No significant differences were observed in D30 white blood cells, red blood cells, hemoglobin, hematocrit, mean corpuscular volume, mean corpuscular hemoglobin, mean corpuscular hemoglobin concentration, red blood cell distribution, neutrophils, monocytes, eosinophils, basophils, total cholesterol, triglycerides, high-density lipoproteins (HDL), low-density lipoproteins (LDL), non-HDL cholesterol, very LDL, LDL/HDL ratio, glucose, blood urea nitrogen (BUN), creatinine (Crn), estimated glomular filtration rate, BUN/Crn, sodium, potassium, chloride, carbon dioxide, albumin (ALB), globulin (GLOB), ALB/GLOB ratio, total bilirubin, alkaline phosphatase, aspartate aminotransaminase, or alanine aminotransaminase. Additionally, chi-square analysis revealed no significant differences between treatment groups in self-reported frequency or severity of dizziness, headache, tachycardia, heart palpitations, shortness of breath, nervousness, blurred vision, or other side effects after 30-days of supplementation.

**Conclusions**: Dietary supplement with 225 mg of liposomal ashwagandha was not associated with clinically significant changes in cell blood counts, serum clinical markers, or reported frequency or severity of side effects. These findings suggest that ashwagandha supplementation was well tolerated in healthy individuals.

**Acknowledgments**: This study was funded as a fee-for-service project awarded to the Human Clinical Research Facility at Texas A&M University from Specnova LLC (Tysons Corner, VA, USA).

**Sex Differences in Perceived Protein Needs, Perceived Protein Intake, and Actual Protein Intake in Collegiate Olympic Weightlifters**

Patrick S. Harty^a^, Harry P. Cintineo^a^, Kyle L. Sunderland^a^, Andrew R. Jagim^b^,Chad M. Kerksick^a^

^a^Exercise and Performance Nutrition Laboratory, Department of Kinesiology, Lindenwood University, St. Charles, MO, USA; ^b^Sports Medicine, Mayo Clinic Health System, La Crosse, WI, USA

Corresponding author: pharty@lindenwood.edu

**Background**: Ensuring appropriate protein intake is vital to help support recovery and increase muscle mass and strength in resistance-trained athletes. However, many athletes fail to consume adequate protein to meet their training demands. Previous research has shown that female athletes are at particular risk of nutritional deficiencies due to inadequate energy and macronutrient consumption, due in part to insufficient nutrition knowledge. Therefore, the purpose of this study was to examine sex-specific nutrition knowledge, perceptions of protein needs and intakes, and compare actual protein intake to evidence-based guidelines from the International Society of Sports Nutrition (ISSN) in a population of collegiate Olympic-style weightlifters.

**Methods**: Collegiate Olympic-style weightlifters (Males: *n* =8, Mean± SD; 19.8 ± 0.7 years, 172.1 ± 5.0 cm, 83.1 ± 17.1 kg, 20.3 ± 5.1 %BF; Females: *n* =12, 20.5 ± 1.9 years, 163.9 ± 5.1 cm, 75.7 ± 13.9 kg, 32.4 ± 5.7% BF) reported their perceived protein needs (PN) and current perceived protein intake (PI) via electronic questionnaire. Average actual protein intake (AI) was determined via 24-hour dietary recalls on two weekdays and one weekend day using the Automated Self-Administered Dietary Assessment Tool (ASA24), which uses a validated multiple-pass approach. Because perceived and actual nutrition data were not normally distributed, a non-parametric approach was used for all analyses. Wilcoxon signed-rank tests were computed within each sex to compare between PN and PI as well as between PI and AI. Sex-specific actual relative protein intakes were compared via the Mann-Whitney U-test. All nutritional results are reported as Median ± IQR. The threshold of significance was set at *p* < 0.05.

**Results**: No differences were detected between PN (200 ± 61 g/d) and PI (180 ± 48 g/d, *p* = 0.672) or between PI (180 ± 48 g/d) and AI (212 ± 58 g/d, *p* = 0.313) in males. However, PN (135 ± 43 g/d) and PI (106 ± 46 g/d, *p* = 0.004) were significantly different in females, though no differences were identified between the females’ PI (106 ± 46 g/d) and AI (107 ± 56 g/d, *p* = 0.380). Relative AI of all males (2.47 ± 0.9 g/kg/d) aligned with or exceeded ISSN recommendations and was significantly greater than the females (1.47 ± 0.9 g/kg/d, *p* = 0.007). Five of the 12 females consumed less than the minimum recommended threshold of 1.4 g/kg/d.

**Conclusions**: Sex differences in relative protein intake and beliefs surrounding protein intake were identified in this population. In contrast to the male lifters, females consumed significantly less protein and believed they consumed less than they needed. 5 of the 12 females consumed less protein than the minimum recommended threshold of 1.4 g/kg/d. Additional targeted nutrition education is warranted in this population.

**Effects of Liposomal Ashwagandha Supplementation on Markers of Health and Cognitive Function in Healthy Adults: COMPASS Cognitive Function Assessment II**

Sarah Johnson^a^, Megan Leonard^a^, Broderick Dickerson^a^, Drew E. Gonzalez^a^,Landry Estes^a^, Dante Xing^a^, Victoria Jenkins^a^, Joungbo Ko^a^, Choongsung Yoo^a^,Martin Purpura^b^, Ralf Jäger^b^, Ryan Sowinski^a^, Christopher J. Rasmussen^a^, Richard B. Kreider^a^*

^a^Exercise & Sport Nutrition Lab, Texas A&M University, College Station, TX 77843, USA; ^b^Increnovo LLC, Whitefish Bay, WI 53217, USA

*Corresponding Author: rbkreider@tamu.edu

**Background**: Ashwagandha has been used in traditional Ayurvedic medicine as an adaptogen to reduce stress, improve memory and cognitive function, and improve exercise performance. We recently reported that acute ingestion of ashwagandha (400 mg) improved selected measures of executive function, helped sustain attention, and increased short-term/working memory. The purpose of this study was to determine if 30 days of ashwagandha supplementation improves markers of health and/or cognitive function in healthy adults.

**Methods**: 59 men and women (22.7 ± 7 yrs., 74.9 ± 16 kg, 26.2 ± 5 BMI) fasted for 12 hours, donated a fasting blood sample, and were administered the COMPASS cognitive function test battery (i.e. Word Recall, Stroop Task, and the Corsi Block Task Test). In a randomized and double-blind manner, participants were then administered 225 mg of a placebo (Gum Arabic, PLA) or ashwagandha (*Withania somnifera*) root and leaf extract coated with a liposomal covering (NooGandha®, Specnova LLC, Tysons Corner, VA, USA, ASH). After 60-min, participants repeated cognitive assessments. Participants continued supplementation (225 mg/d) for 30-days and then returned to the lab to repeat the experiment. Data were analyzed by a General Linear Model (GLM) univariate analyses with repeated measures and pairwise comparisons of mean changes from baseline with 95% Confidence Intervals (CI).

**Results**: Multivariate analysis of changes in Word Recall Task Test variables from baseline revealed a significant time (*p* < 0.004, $\eta_{p}^{2}$ = 0.077, moderate effect) with no significant treatment x time effects (*p* = 0.750, $\eta_{p}^{2}$ = 0.0106, small effect). Univariate analysis revealed significant time effects in Total Attempts (p = 0.001), Correct Attempts (p = 0.005), Recalled Attempts (p = 0.001), and Recalled Correct (*p* = 0.001) with no significant interaction effects. Pairwise analysis revealed that 30-day (D30)-Post Recalled Correct values tended to be higher with ASH supplementation (*p* = 0.094) with Recalled Attempts and Correct Recalled decreasing from baseline in the PLA group. Multivariate analysis of changes in Stroop Task Test variables from baseline revealed a significant time (*p* < 0.011, $\eta_{p}^{2}$ = 0.088, moderate effect) with no significant treatment x time effects (*p* = 0.594, $\eta_{p}^{2}$ = 0.048, small effect). Univariate analysis revealed a significant time effects in Overall Reaction Time (*p* = 0.001), Correct Reaction Time (*p* = 0.001), Congruent Overall Reaction Time (*p* = 0.001), Incongruent Overall Reaction Time (*p* = 0.001), Congruent Correct Reaction Time (*p* = 0.001) while Congruent Percent Correct (*p* = 0.059) and Incongruent Correct Reaction Time tended to differ (*p* = 0.062) with no significant interaction effects. Pairwise analysis revealed that D0-Post (0.8 [−.0.014, 1.6], *p* = 0.064) and D30-Post (1.0 [−0,17, 2.1], *p* = 0.092) tended to be higher with ASH supplementation. Multivariate analysis of changes in Corsi Task Test variables from baseline revealed no significant time (*p* < 0.688, $\eta_{p}^{2}$ = 0.028, small effect) or group x time effects (*p* = 0.671, $\eta_{p}^{2}$ = 0.028, small effect). Likewise, no significant univariate time or group x time effects or pairwise comparison effects were observed in Corsi Task Test results.

**Conclusions**: Results revealed evidence that ASH supplementation improved some markers of cognitive function, memory, and reaction time particularly after D30 of supplementation.

**Acknowledgments**: This study was funded as a fee-for-service project awarded to the Human Clinical Research Facility at Texas A&M University from Specnova, LLC (Tysons Corner, VA, USA).

**Effects of Dietary Supplementation of a Microalgae Extract Containing Fucoxanthin Combined with Guarana on Cognitive Function and Gaming Performance III: Light Reaction Test**

Broderick Dickerson^a^, Ryan Sowinski^a^, Megan Leonard^a^, Drew Gonzalez^a^,Jacob Kendra^a^, Victoria Martinez^a^, Kay Nottingham^a^, Choongsung Yoo^a^, Dante Xing^a^, Joungbo Ko^a^, Sarah Johnson^a^, Landry Estes^a^, Jonathan Maury^b^, Rémi Pradelles^b^,Mark Faries^a,c^, Wesley Kephart^d^, Christopher J. Rasmussen^a^, Richard B. Kreider^a^*

^a^Exercise & Sport Nutrition Lab, Texas A&M University, College Station, TX 77843, USA; ^b^Microphyt, Research & Development Department, Baillargues, Mudaison, 34,670, FRA; ^c^Texas A&M AgriLife Extension, Texas A&M University, College Station, TX 77843, USA; ^d^Department of Kinesiology, University of Wisconsin – Whitewater, Whitewater, WI 53190, USA

*Corresponding author: rbkreider@tamu.edu

**Background**: E-sports competitive gaming requires selective visual attention, short-term memory or task switching, and an ability to sustain psychomotor performance over time. Fucoxanthin is a carotenoid, found in specific microalgae varieties, such as *Phaeodactylum tricornutum* (*PT*), that have been reported to possess neuroprotective and nootropic effects through its anti-inflammatory and antioxidant activities. This study evaluated whether acute and 30-day supplementation of a microalgae extract from *PT* combined with Guarana affects cognitive function in gamers.

**Methods**: In a double-blind, placebo-controlled manner, 61 experienced gamers (21.7 ± 4.1 years, 73 ± 13 kg, 24.2 ± 3.6 kg/m^2^) were randomly assigned to ingest a placebo (PL); a low-dose (LD) supplement containing 440 mg of *PT* extract including 1% fucoxanthin + 500 mg of Guarana (GamePhyt™, Microphyt, Baillargues, FR) or a high-dose (HD) supplement containing 880 mg of *PT* extract + 500 mg of Guarana for 30-days. A light-tracking reaction performance test (NeuroTracker) was administered on Day 0 pre-supplementation, 15-min post-supplementation, and after 60 minutes of competitive gameplay with participants’ most played video game. Participants were supplemented for 30 days, then repeated pre-supplementation, gaming, and post-gaming tests. Data were analyzed by General Linear Model (GLM) univariate and multivariate analyses with repeated measures and mean changes from baseline with 95% confidence intervals.

**Results**: Significant time (*p* < 0.001, η_p_^2^ = 0.159, large effect) with no significant group x time interaction effects (*p* = 0.531, η_p_^2^ = 0.032, small effect) were observed. Start-speed increased in all groups over time. Analysis of mean changes showed some group differences in start speed and percentage of correct targets. Day 0, start speed increased from baseline at 15-Post-Supp in PL and LD, but not HD. Post-Game start speed increased in all groups with LD higher than HD, but no different than PL. Day 30, Pre-start speed was better maintained in LD than HD, while percent targets correct decreased in HD, compared to baseline. Post-Game start speed was higher than the baseline in all groups. Post-Game start speed and percent targets correct were higher in LD than HD, but no different than PL. Gaming performance scores (N = 57) were normalized and expressed as Z scores (PL = 18, LD = 19, HD = 20). Pearson Correlations between gaming Z scores and light reaction scores on Day 0, pre-supplement showed a very weak (r < 0.2) negative correlation in PL [N = 18; r = −0.011; p = 0.966], weak (0.2 < r < 0.3) in LD [N = 19; r = 0.356; *p* = 0.135], and negative weak in HD [N = 20; r = −0.294; *p* = 0.209] with post-supplement scores having a very weak correlation in PL [N = 18; r = 0.097; *p* = 0.703], weak in LD[N = 19; r = 0.288; *p* = 0.233], and negative weak in HD [N = 20; r = −0.314; *p* = 0.178]. Day 30, pre-supplement score correlations were very weak in PL [N = 18; r = 0.168; *p* = 0.505], weak in LD [N = 19; r = 0.281; *p* = 0.244], and negative weak in HD [N = 20; r = −0.116; *p* = 0.485], while post-supplement correlations were very weak in PL [N = 18; r = 0.098; *p* = 0.700], and negative weak in LD [N = 19; r = −0.111; *p* = 0.651] as well as HD [N = 20; r = −0.004; *p* = 0.986]. Correlations between gaming Z scores and other cognitive measures (i.e. PEBL tasks) were weak.

**Conclusion**: These findings suggest a slight improvement in performance in both high and low doses, although not significantly different from that of the placebo group. Further, there does not seem to be any meaningful correlations between gaming performance Z scores, light reaction test performance, or PEBL (Sternberg, Go/No-Go, Vigilance) tasks.

**Acknowledgments**: This study was funded by MicroPhyt (Baillargues, FR) as a fee-for-service project to the Human Clinical Research Facility at Texas A&M University and conducted by the Exercise & Sport Nutrition Lab.

**Examining Limb Asymmetry and Digit Ratios in Collegiate Female Soccer Players**

Maxine Furtado Mesa, Jeffrey R. Stout, David H. Fukuda

University of Central Florida, Orlando, FL, USA

Corresponding author: maxine.furtado@ucf.edu

**Background**: Bilateral asymmetries can lead to muscle injuries in soccer players (Croisier et al., 2008). Studies have shown uneven gluteal muscle development in soccer players (Sanchis-Moysi et al., 2011) and associated injuries (Hanson et al., 2008). However, the influence of anthropometric characteristics on player success has not been fully explored. Studies on the ratio between the lengths of the index (2D) and ring (4D) fingers (2D:4D) suggest that a low digit ratio could signify potential sports ability due to prenatal testosterone exposure (Manning and Taylor, 2001). In youth soccer players, the 2D:4D ratio has been shown to predict changes in maximal oxygen consumption and muscular strength (Nobari et al., 2021). Thus, it becomes critical to understand factors that could contribute to athletic performance, including anthropometric indicators such as the 2D:4D ratio. This study aims to evaluate the anthropometric measurements of female collegiate soccer players, focusing primarily on potential limb dominance and its potential implications for training and injury prevention.

**Methods**: Thirty participants volunteered to participate in the study. Anthropometric measurements were taken for both the dominant and nondominant sides, including second and fourth digit ratios (2D: 4D) ratios, skinfold measurements, limb and trunk circumference, and bone diameter. Nine skinfolds were measured using the Lange Skinfold Caliper and 15 girths and eight widths were measured with a standard tape measure and a sliding Harpenden anthropometer. Body composition components were calculated, including fat, muscle, and residual mass, to compare with pre-season maximal oxygen consumption (VO2max). VO2max was derived from pre-season Yo-Yo Intermittent Recovery Test Level 1 (YYIRT) scores using the following equation: VO_2max_ =YYIRT distance (m)×0.0084 +36.4 (Bansgbo et al., 2008). Wilcoxon signed-rank tests were used to compare anthropometric values between dominant and nondominant limbs. Spearman’s rho correlations were used to examine the relationships between anthropometric variables and estimated VO_2max_.

**Results**: There were no significant differences between the circumferences of the dominant and non-dominant proximal, medial and distal thighs, knees, calves, ankles, and diameters of the dominant and non-dominant knees and ankles. There was a significant correlation between VO_2max_ and right 2D:4D (rs = −.426, p = .021). No significant correlations were found between VO_2max_ and muscle mass, fat mass, residual mass, and left 2D: 4D. No significant correlations were found between VO_2max_ and skinfold measurements, circumference measurements. No significant correlations were found between the right 2D:4D, skinfold, and diameter measurements.

**Conclusions**: These results suggest that bilateral asymmetry was not observed in this group of college soccer players. Interestingly, a correlation was found between the digit ratio (2D:4D) and maximum oxygen consumption (VO_2max_) in collegiate female soccer players. This correlation indicates a potential relationship between prenatal sex hormone exposure and athletic performance (Paul et al., 2006).

**Effects of Dietary Supplementation of a Microalgae Extract Containing Fucoxanthin Combined with Guarana on Cognitive Function and Gaming Performance II: Profile of Mood States**

Victoria Martinez^a^, Ryan Sowinski^a^, Megan Leonard^a^, Broderick Dickerson^a^,Drew Gonzalez^a^, Jacob Kendra^a^, Kay Nottingham^a^, Choongsung Yoo^a^, Dante Xing^a^, Joungbo Ko^a^, Sarah Johnson^a^, Landry Estes^a^, Jonathan Maury^b^, Rémi Pradelles^b^,Mark Faries^a,b^, Wesley Kephart^d^, Christopher J. Rasmussen^a^, Richard B. Kreider^a^*

^a^Exercise & Sport Nutrition Lab, Texas A&M University, College Station, TX 77843, USA; ^b^Microphyt, Research & Development Department, Baillargues, Mudaison, 34,670, FRA; ^c^Texas A&M AgriLife Extension, Texas A&M University, College Station, TX 77843, USA; ^d^Department of Kinesiology, University of Wisconsin – Whitewater, Whitewater, WI 53190, USA

*Corresponding author: rbkreider@tamu.edu

**Background**: Esports competitive gaming requires selective visual attention, short-term memory or task switching, and an ability to sustain psychomotor performance over time. Fucoxanthin is a carotenoid, found in specific microalgae varieties, such as *Phaeodactylum tricornutum* (*PT*), that have been reported to possess neuroprotective and nootropic effects through its anti-inflammatory and antioxidant activities. This study evaluated whether acute and 30-day supplementation of a microalgae extract from *PT* combined with Guarana affects cognitive function in gamers.

**Methods**: In a double-blind, placebo-controlled manner, 61 experienced gamers (21.7 ± 4.1 years, 73 ± 13 kg, 24.2 ± 3.6 kg/m^2^) were randomly assigned to ingest a placebo (PL); a low-dose (LD) supplement containing 440 mg of *PT* extract including 1% fucoxanthin + 500 mg of Guarana (GamePhyt™, Microphyt, Baillargues, FR) or a high-dose (HD) supplement containing 880 mg of *PT* extract + 500 mg of Guarana for 30-days. Profile of Mood States (POMS) were administered on Day 0 before supplementation, 15-min post-supplementation, and after 60 minutes of competitive gameplay with participants’ most played video game. Participants continued supplementation for 30 days then repeated pre-supplementation and post-gaming POMS. Data were analyzed by General Linear Model (GLM) multi- and univariate analyses with repeated measures and mean changes from baseline with 95% confidence intervals.

**Results**: Multivariate analysis of the overall POMS assessment revealed significant time (*p* < 0.001, η_p_^2^ = 0.083) but no group x time interaction effects (*p* = 0.836, η_p_^2^ = 0.027). The POMS assessment covered several domains such as Tension, Depression, Anger, Fatigue, Confusion, and Vigor. When broken down into individual domains a significant time but not group x time effect was found in Tension (p < 0.001, ηp2 = 0.141; p = 0.604, η^2^ = 0.036); Depression (p = 0.001, η^2^ = 0.103; p = 0.561, η^2^ = 0.061), Fatigue (p = 0.006, η^2^ = 0.053; p = 0.521, η^2^ = 0.033), Confusion (p < 0.001, η^2^ = 0.067; p = 0.488, η^2^ = 0.033) and Vigor (p = 0.003, η^2^ = 0.060; p = 0.885, η^2^ = 0.027). Analysis of individual items showed positive effects in all groups for tense, relaxed, unhappy, lonely across all time points. Day 30 Post Game efficient scores were significantly increased for the LD group. The HD group showed improvement in uneasy, restless, sorry for things done, sad, and gloomy across all time points. Day 30 Post Game listless scores also decreased in the HD group. Day 0 Post-Game vigor scores significantly declined in PL and LD. Day 30 Pre- and Post-Game vigor scores were also better maintained with HD compared to PL and LD.

**Conclusion**: Results provide some evidence that microalgae extract from PT combined with Guarana may support mood domains after acute and 30 days of supplementation at both doses studied.

**Acknowledgments**: This study was funded by MicroPhyt (Baillargues, FR) as a fee-for-service project to the Human Clinical Research Facility at Texas A&M University and conducted by the Exercise & Sport Nutrition Lab.

**Effects of Liposomal Ashwagandha Supplementation on Markers of Health and Cognitive Function in Healthy Adults: COMPASS Cognitive Function Assessment I**

Megan Leonard^a^, Broderick Dickerson^a^, Drew E. Gonzalez^a^, Sarah Johnson^a^,Landry Estes^a^, Dante Xing^a^, Victoria Jenkins^a^, Joungbo Ko^a^, Choongsung Yoo^a^,Martin Purpura^b^, Ralf Jäger^b^, Ryan Sowinski^a^, Christopher J. Rasmussen^b^,Richard B. Kreider^a^*

^a^Exercise & Sport Nutrition Lab, Texas A&M University, College Station, TX 77843, USA; ^b^Increnovo LLC, Whitefish Bay, WI 53217

*Corresponding Author: rbkreider@tamu.edu

**Background**: Ashwagandha has been used in traditional Ayurvedic medicine as an adaptogen to reduce stress, improve memory and cognitive function, and improve exercise performance. We recently reported that acute ingestion of ashwagandha (400 mg) improved selected measures of executive function, helped sustain attention, and increased short-term/working memory. The purpose of this study was to determine if 30 days of ashwagandha supplementation improves markers of health and/or cognitive function in healthy adults.

**Methods**: 59 men and women (22.7 ± 7 yrs., 74.9 ± 16 kg, 26.2 ± 5 BMI) fasted for 12 hours, donated a fasting blood sample, and were administered the COMPASS cognitive function test battery (Word Recognition, Choice Reaction Time Task, Picture Recall, and Digit Vigilance Task). In a randomized and double-blind manner, participants were then administered 225 mg of a placebo (Gum Arabic, PLA) or ashwagandha (*Withania somnifera*) root and leaf extract coated with a liposomal covering (NooGandha®, Specnova LLC, Tysons Corner, VA, USA, ASH). After 60-min, participants repeated cognitive assessments. Participants continued supplementation (225 mg/d) for 30-days and then returned to the lab to repeat the experiment. Data were analyzed by a General Linear Model (GLM) univariate analyses with repeated measures and pairwise comparisons of mean changes from baseline with 95% Confidence Intervals (CI).

**Results**: Multivariate analysis of changes in Word Recognition from baseline revealed a significant time (*p* < 0.001, $\eta_{p}^{2}$ = 0.081, moderate effect) with no significant treatment x time effects (*p* = 0.654, $\eta_{p}^{2}$ = 0.035, small effect). Univariate analysis revealed a significant time effect in Percent Yes Correct responses (*p* = 0.002) and No Response Time (*p* = 0.004). Pairwise comparison revealed that Percent Yes Correct responses increased after 30-days (D30) with PLA, while Reaction Times were generally decreased over time with ASH supplementation. However, no significant differences were observed between the groups. Multivariate analysis of changes in Choice Reaction Time variables from baseline revealed a significant time (*p* < 0.033, $\eta_{p}^{2}$ = 0.066, moderate effect) with no significant treatment x time effects (*p* = 0.427, $\eta_{p}^{2}$ = 0.042, small effect). Univariate analysis revealed a significant time effect in Percent Picture Recall Percent Correct (*p* = 0.014) with trends observed in percent Yes and No Correct Responses. No significant differences were observed between the ASH and PLA groups, although the Percent Correct increased over time, while Reaction Times decreased with PLA ingestion. Multivariate analysis of changes in Picture Recall variables from baseline revealed a significant time (*p* < 0.033, $\eta_{p}^{2}$ = 0.066, moderate effect) with no significant treatment x time effects (*p* = 0.424, $\eta_{p}^{2}$ = 0.042, small effect). Univariate analysis revealed a significant time effect in Percent Picture Recall Correct (*p* = 0.014) with trends observed in Percent Yes (*p* = 0.073) and No Correct responses (*p* = 0.073). No significant univariate interaction effects were observed between treatments. However, pairwise analysis revealed that D0-Post and D30-Pre reaction times were faster values ASH ingestion. Multivariate analysis of changes in Digital Vigilance Task Test variables from baseline revealed a significant time (*p* < 0.004, $\eta_{p}^{2}$ = 0.046, moderate effect) with no significant treatment x time effects (*p* = 0.804, $\eta_{p}^{2}$ = 0.010, small effect). Univariate analysis revealed a significant time effect in the Correct Reaction Time (*p* = 0.002) with no significant interaction effects. Pairwise analysis revealed that D0-Post Reaction Times increased while False Alarms were lower with ASH ingestion, while D30-Post Percent Correct decreased from baseline with PLA ingestion.

**Conclusions**: Results revealed evidence that ASH supplementation improved markers of cognitive function, memory, and reaction time particularly after D30 of supplementation.

**Acknowledgments**: This study was funded as a fee-for-service project awarded to the Human Clinical Research Facility at Texas A&M University from Specnova LLC (Tysons Corner, VA, USA).

**Effects of Liposomal Ashwagandha Supplementation on Markers of Mood**

Landry Estes^a^, Megan Leonard^a^, Broderick Dickerson^a^, Drew E. Gonzalez^a^,Sarah Johnson^a^, Dante Xing^a^, Victoria Jenkins^a^, Joungbo Ko^a^, Choongsung Yoo^a^,Martin Purpura^b^, Ralf Jäger, FISSN^b^, Ryan Sowinski^a^, Christopher J. Rasmussen^a^,Richard B. Kreider, FISSN^a^*

^a^Exercise & Sport Nutrition Lab, Texas A&M University, College Station, TX 77843, USA; ^b^Increnovo LLC, Whitefish Bay, WI 53217

*Corresponding Author: rbkreider@tamu.edu

**Background**: Ashwagandha has been used in traditional Ayurvedic medicine as an adaptogen to reduce stress, improve memory and cognitive function, and improve exercise performance. We recently reported that acute ingestion of ashwagandha (400 mg) improved selected measures of executive function, helped sustain attention, and increased short-term/working memory. The purpose of this study was to determine if 30 days of ashwagandha supplementation improves markers of health and/or cognitive function in healthy adults.

**Materials and Methods**: 59 men and women (22.7 ± 7 yrs., 74.9 ± 16 kg, 26.2 ± 5 BMI) fasted for 12 hours, donated a fasting blood sample, and were administered the Profile of Mood States inventory (POMS). In a randomized and double-blind manner, participants were then administered 225 mg of a placebo (maltodextrin, PLA) or a liposomal ashwagandha (*Withania somnifera*) root and leaf extract (NooGandha®, Specnova LLC, Tysons Corner, VA, USA, ASH). After 60-min, participants repeated cognitive assessments. Participants continued supplementation (400 mg/d) for 30-days and then returned to the lab to repeat the experiment. Individual POMS items, scored dimensions, and total mood disturbance scores were analyzed by a General Linear Model (GLM) univariate analyses with repeated measures and pairwise comparisons of mean changes from baseline with 95% Confidence Intervals (CI).

**Results**: Multivariate analysis of changes in POMS scored dimensions from baseline revealed a significant time (*p* < 0.001, $\eta_{p}^{2}$ = 0.148, large effect) with no significant treatment x time effects (*p* = 0.108, $\eta_{p}^{2}$ = 0.049, moderate effect). Univariate analysis revealed a significant interaction in fatigue (*p* = 0.033, $\eta_{p}^{2}$ = 0.061, moderate effect). Pairwise comparison revealed that fatigue values after 30-days (D30) decreased from baseline (D0) with ASH supplementation with D30-D0 values significantly lower than group PLA (−2.57 [−4.6, −0.5], *p* = 0.016), while D30-Post values tended to be lower with ASH (−1.72 [−3.7, 0.3], *p* = 0.092). Analysis of individual POMS items revealed that those supplementing with ASH had significantly higher D0-Post Ready-to-Fight sores (0.46 [0.015, 0.9], *p* = 0.043) with significantly lower D30-Pre ratings of Resentful (−0.28, [−0.5, −0.008], *p* = 0.044) and D30-Post ratings of Sad (−0.21 [−.42, −.004], *p* = 0.046), and Panicky (−0.38 [−0.72, −0.05], *p* = 0.027) scores. Trends were also observed with those in the ASH group reporting lower D30-Pre Worn Out (*p* = 0.068), Unhappy (*p* = 0.089), Grouchy (*p* = 0.059) scores and D30-Post Tense (p = 0.062), On-Edge (*p* = 0.096), Grouchy (*p* = 0.078), annoyed (*p* = - 0.089) and Miserable (*p* = 0.073) scores while perceptions of D0-Post Bushed (*p* = 0.076), D30-Pre Muddled (*p* = 0.090) and D30-Post Sympathetic (*p* = 0.076) and Worthless (*p* = 0.096) tended to be higher.

**Conclusions**: Results indicate that liposomal ashwagandha supplementation improved perceptions of fatigue, and feelings of anxiety, sadness, ready-to-fight and resentfulness.

**Acknowledgments**: This study was funded as a fee-for-service project awarded to the Human Clinical Research Facility at Texas A&M University from Specnova, Inc. (Boca Raton, FL, USA).

**Effects of Dietary Supplementation of a Microalgae Extract Containing Fucoxanthin Combined with Guarana on Cognitive Function and Gaming Performance I: Game Performance**

Ryan Sowinski^1^a Megan Leonard^a^, Broderick Dickerson^a^, Drew Gonzalez^a^,Jacob Kendra^a^, Victoria Martinez^a^, Kay Nottingham^a^, Choongsung Yoo^a^, Dante Xing^a^, Joungbo Ko^a^, Sarah Johnson^a^, Landry Estes^a^, Jonathan Maury^a^, Rémi Pradelles^b^,Mark Faries^a,c^, Wesley Kephart^d^, Christopher J. Rasmussen^a^, Richard B. Kreider^a^*

^a^Exercise & Sport Nutrition Lab, Texas A&M University, College Station, TX 77843, USA; ^b^Microphyt, Research & Development Department, Baillargues, Mudaison, 34,670, FRA; ^c^Texas A&M AgriLife Extension, Texas A&M University, College Station, TX 77843, USA; ^d^Department of Kinesiology, University of Wisconsin – Whitewater, Whitewater, WI 53190, USA

*Corresponding author: rbkreider@tamu.edu

**Background**: Esports competitive gaming requires selective visual attention, short-term memory or task switching, and an ability to sustain psychomotor performance over time. Fucoxanthin is a carotenoid, found in specific microalgae varieties, such as *Phaeodactylum tricornutum* (*PT*), that have been reported to possess neuroprotective and nootropic effects through its anti-inflammatory and antioxidant activities. This study evaluated whether acute and 30-day supplementation of a microalgae extract from *PT* combined with Guarana affects cognitive function in gamers.

**Methods**: In a double-blind, placebo-controlled manner, 61 experienced gamers (21.7 ± 4.1 years, 73 ± 13 kg, 24.2 ± 3.6 kg/m^2^) were randomly assigned to ingest a placebo (PL); a low-dose (LD) supplement containing 440 mg of *PT* extract including 1% fucoxanthin + 500 mg of Guarana (GamePhyt™, Microphyt, Baillargues, FR), or a high-dose (HD) supplement containing 880 mg of *PT* extract + 500 mg of Guarana for 30-days. Participants performed 60 minutes of competitive gameplay with their most played video game on Day 0 and after 30 days of supplementation. Scores were taken from five video games (i.e. Call of Duty, Super Smash Brothers, Overwatch, League of Legends, and Valorant). Gaming scores were normalized using ‘Min-Max Scaling’, which rescales data points between 0 and 1, using the lowest and highest values, establishing a relative range. Each variable (e.g. kills, deaths, etc.) was recalculated (Xnew = (X – Xmin)/ (Xmax – Xmin)) and assigned a new value (Xnew), using the highest (Xmax) and lowest (Xmin) scores for that game/mode. Every round/match score was rescaled and averaged to create a single ‘round performance score’. These scores, from the same session, were then averaged into a ‘performance score’ for the whole session. The goal of normalizing the gaming scores was to yield relative values, allowing for comparison across game modes, titles, and platforms. Data were analyzed by General Linear Model (GLM) univariate and multivariate analyses with repeated measures and mean changes from baseline with 95% confidence intervals.

**Results**: Out of 61 participants, 3 had incomplete or irretrievable game data. Of the five games (N = 58), Call of Duty (N = 17), Super Smash Brothers (N = 13), Overwatch (N = 7), League of Legends (N = 11), and Valorant (N = 10), Call of Duty and Super Smash Brothers scores were from varying gameplay modes, 7 and 3, respectively. Another person was dropped for playing a game mode that was unable to be scaled. Thus, a total of 57 scores were able to be normalized and expressed as Z scores (PL = 18, LD = 19, HD = 20). Analysis of the normalized scores showed no time (*p* = 0.536, η_p_^2^ = 0.007) or group x time effects (*p* = 0.468, η_p_^2^ = 0.028). One-way ANOVA of mean changes showed no differences among groups (PL 0.0091 ± 0.087; LD −0.0428 ± 0.174; HD −0.0002 ± 0.133, *p* = 0.468, η_p_^2^ = 0.028) or between PL and LD (0.052 [−0.038, 0.142], *p* = 0.254) or HD (0.009 [−0.080, 0.098], *p* = 0.836) scores. Similarly, percent change in Z scores showed no group differences (PL 1.56 ± 28.9; LD 3.53 ± 52.4; HD 8.76 ± 44.7%, *p* = 0.868, η_p_^2^ = 0.005) or between PL and LD (−1.96% [−30.5, 26.6], *p* = 0.891) or HD (−7.20% [−35.4, 21.0], *p* = 0.611).

**Conclusion**: Normalized values allowed for a cumulative analysis of gaming performance data. However, these findings indicate that while mean percentage changes in performance appeared to be more favorable with LD and HD, they were not significant due to a large amount of variability. Therefore, performance was consistent among groups.

**Acknowledgments**: This study was funded by MicroPhyt (Baillargues, FR) as a fee-for-service project to the Human Clinical Research Facility at Texas A&M University and conducted by the Exercise & Sport Nutrition Lab.

**Creatine Supplementation Improves Forcible Entry Performance in Career Firefighters.**

Kaia Elstad^a^, Joel Luedke^a^, Jaime Salvador^a^, Ward Dobbs^a^, Thomas Almonroeder^c^,Chad M. Kerksick^b,d^, Adam Markert^e^, Andrew R. Jagim^a,b^

^a^Exercise & Sport Science Department, University of Wisconsin – La Crosse, La Crosse, WI, USA; ^b^Sports Medicine, Mayo Clinic Health System, La Crosse, WI, USA; ^c^Department of Physical Therapy, Trine University, Angola, IN, USA; ^d^Exercise and Performance Nutrition Laboratory, Department of Kinesiology, Lindenwood University, St. Charles, MO, USA; ^e^La Crosse Fire Department, La Crosse, WI, USA

Corresponding author: jagim.andrew@mayo.edu

**Background**: Firefighters regularly perform high-intensity, functional tasks as part of their profession and therefore may benefit from the increased availability of short-term energy. Creatine supplementation has been shown to positively impact various aspects of physical performance and work capacity. However, less information is available regarding the effects of creatine supplementation on occupational performance in firefighters. The purpose of this study was to investigate, the effects of creatine supplementation on occupational performance in firefighters.

**Methods**: Twenty-nine career firefighters (mean± SD; 34.4 ± 8.2 years, 1.82 ± 0.07 m, 88.8 ± 12.3 kg, 17.5 ± 5.9%BF) were randomized into one of the two supplementation groups to ingest A) 25 grams of Whey Protein + 25 grams of Carbohydrate (Control Group); or B) 25 grams of Whey Protein + 25 grams of Carbohydrate + 5 grams of Creatine (Experimental Group) in a double-blind fashion, each day. At baseline and again at 18-24 days post-baseline testing, participants completed a battery of tests to evaluate changes in occupational performance. Throughout the supplementation period, firefighters were allowed to exercise per their normal routine. The battery of tests included a hose line advance, rescue (body drag), stair climb, and forcible entry. Firefighters also completed a maximal aerobic speed (MAS) test on a cycle ergometer the next day. All results are reported as Mean ± SD. The threshold of significance was set at *p* < 0.05.

**Results**: There was a significant group x time interaction for rescue, stair climb and forcible entry. Pairwise comparisons indicated that the control group recorded a greater reduction in completion time for the rescue (Control: −.135, 95% CI: −0.948, 0.678 vs. Creatine: 1.31, 95% CI: 0.497, 2.12 sec, *p* = 0.017), and the stair climb compared to creatine group (Control: −3.53, 95% CI: −6.01, −1.05 vs. Creatine: 0.15, 95% CI: −2.33, 2.63 sec, *p* = 0.041). There was a significant group x time interaction for forcible entry, with the creatine group recording a greater improvement in time to completion compared to the control group (Creatine: −1.73, 95% CI: −3.61, 0.15 vs. Control: 1.59, 95% CI: −0.29, 3.47 sec, *p* = 0.01). No significant time effects, or group x time interactions were observed for hose line advance, body drag, or total time to completion. There was a main effect of time for MAS times (*p* < 0.001), with both groups recording faster times during post-testing; however, no significant group x time interaction was observed (*p* = 0.079) (Table 1).

**Conclusions**: The addition of supplemental protein and carbohydrates to the diet of career firefighters throughout a three-week period appears to improve occupational performance in firefighters. The addition of creatine supplementation led to greater improvements in time to completion for forcible entry in firefighters, a task involving high power output. Creatine supplementation did not lead to further improvements in additional measures of occupational performance in firefighters.

**A Dose-Response Study to Examine Paraxanthine’s Impact on Energy Expenditure, Hunger, Appetite, and Lipolysis**

Kristen N. Gross^a^, Leah E. Allen^a^, Anthony M. Hagele^a^, Joesi M. Krieger^a^,Paige J. Sutton^a^, Esther Duncan^a^, Petey Mumford^a^, Ralf Jäger^b,c^, Martin Purpura^b,c^, Chad M. Kerksick^a^*

^a^Exercise and Performance Nutrition Laboratory, Kinesiology Department, College of Science, Technology and Health, Lindenwood University, St. Charles, MO USA; ^b^Increnovo, LLC, Milwaukee, WI USA; ^c^Ingenious Ingredients L.P., Lewisville, TX USA

Corresponding author: ckerksick@lindenwood.edu

**Background**: Caffeine (CAF) administration increases energy expenditure, lipolysis, and perceived feelings of appetite while reducing hunger. It has been speculated that CAF’s efficacy is based on its main metabolite, paraxanthine (PX). The purpose of the study was to investigate if PX administration has beneficial effects on energy expenditure, lipolysis, and perceived feelings of hunger and appetite.

**Methods**: In a randomized, double-blind, placebo-controlled, crossover fashion, 13 males and 8 females (N =21; 26.0 ± 6.4 years, 174.9 ± 11.5 cm, 81.0 ± 15.7 kg, 26.3 ± 3.4 kg/m^2^) consumed a single-dose of placebo (PLA), 100 mg (PX100), 200 mg (PX200), and 300 mg (PX300), of PX (enfinity®, Ingenious Ingredients, L.P., Lewisville, TX, USA). Venous blood samples were collected 0, 30, 60, 90, 120, and 180 minutes (min) after ingestion and were analyzed for glycerol and free fatty acids (FFA). Safety was evaluated with complete blood counts and comprehensive metabolic panels at 0 and 180 min. In addition, resting hemodynamics, metabolic rates and perceptual indicators of hunger, appetite and anxiety were evaluated at all time points. Within-within factorial ANOVA with repeated measures were used to evaluate interaction and main effects on raw and area under the curve (AUC) data. Significant main effects were evaluated using paired sample t-tests.

**Results**: No pre-supplementation differences (p > 0.05) were noted for body mass, energy expenditure, or hemodynamics. The heart rate decreased in PX100 compared to PLA at 60 (p = 0.022) and 180 minutes (p = 0.001) after ingestion while no changes in blood pressure were noted. Hunger VAS ratings in PLA increased at a significantly greater rate 60 and 90 minutes after ingestion when compared to PX200. No other changes in VAS were observed. PX200 was responsible for significantly greater increases in energy expenditure at all time points when compared to PLA. PX200 resulted in 100 ± 116 kcal/180 min increase in calories burned, compared to 4 ± 126 kcal/180 for PLA. Rates of fat oxidation tended to increase to a greater degree at 90 (*p* = 0.056) and 120 minutes (*p* = 0.066) in PX200 compared to placebo, and levels of free fatty acids increased significantly in the PX300 group compared to placebo (*p* < 0.05). No changes in glycerol were observed between groups.

**Conclusion**: Ingestion of a 200 mg dose of paraxanthine increased energy expenditure and was able to effectively blunt the observed increases in hunger when compared to PLA.

**Acknowledgments:**This research was funded by Ingenious Ingredients. The sponsor played no role in collecting the data and analyzing the data.

**Effects of 10 Weeks of Dileucine Supplementation on Body Composition in Resistance Trained Males**

Joesi M. Krieger^a^, Anthony M. Hagele^a^, Joshua Iannotti^a^, Kevin F. Holley^a^, Paige Sutton^a^, Leah Allen^a^, Connor J. Gaige^a^, Logan S. Orr^a^, Ralf Jäger^b,c^, Martin Purpura^b,c^,Chad M. Kerksick^a^*

^a^Exercise and Performance Nutrition Laboratory, Department of Kinesiology, Lindenwood University, St. Charles, MO, USA; ^b^Increnovo, Whitefish Bay, WI USA; ^c^Ingeniuos Ingredients L.P., Lewisville, TX USA

*Corresponding author: ckerksick@lindenwood.edu

**Background**: Absorption of amino acids, particularly the essential amino acids, is known to be a critical factor that influences observed rates of muscle protein synthesis. Further, the appearance of leucine in optimal amounts and at opportune times can further drive changes in muscle protein synthesis rates. Dileucine, a dipeptide composed of two leucine molecules, has shown potential to increase the appearance of leucine and has previously exhibited a greater ability to stimulate increases in muscle protein synthesis when compared to leucine. Currently, no data exists which examines the potential for dileucine supplementation to impact changes in body composition. This study aimed to investigate the impact of 10 weeks of dileucine supplementation in comparison to leucine and placebo supplementation on body composition parameters in resistance trained males.

**Methods**: 32 resistance trained males (28.4 ± 6.0 years, 176.3 ± 7.4 cm, 79.7 ± 12.5 kg, 25.7 ± 3.9 kg/m2, 19.0 ± 5.7% fat) were randomly assigned to ingest in a double-blind fashion and on a daily basis either 2 grams of dileucine monohydrate (RAMPS™, Ingenious Ingredients), 2 grams of leucine, or 2 grams of resistant starch for 10 weeks. In conjunction with the supplementation protocol, participants were assigned to complete a 4 day per week, split-body resistance training program that included resistance-based exercise to target every major muscle group across 3-4 sets of 6-10 repetitions at intensities from 70 – 85% 1RM. After 0, 2, 6, and 10 weeks of supplementation, participants were evaluated for changes in body composition (Hologic Discovery DXA), body water (InBody 570), and dry lean mass. Body composition parameters, including fat-free mass, fat mass, and percentage body fat, were assessed using a 4-compartment model combining DEXA and BIA measurements. Data were analyzed using mixed factorial ANOVAs using SPSS and one-way ANOVA using change scores from baseline. Data in presented as means ± SD.

**Results**: Changes in body mass after 10 weeks tended to be different between the three supplementation groups (p = 0.08), while no individual pairwise comparisons were statistically significant (p > 0.05). No significant group x time differences were realized for DXA fat-free mass (LEU: 1.22 ± 0.38 kg, DILEU: 1.15 ± 2.48 kg, PLA: 2.15 ± 3.06 kg, *p* = 0.71), DXA fat mass (LEU: 0.26 ± 1.13 kg, DILEU: 0.94 ± 1.30 kg, PLA: −0.17 ± 1.38 kg, *p* = 0.39), DXA fat percentage (LEU: 0.08 ± 1.11%, DILEU: 0.75 ± 1.35%, PLA: −0.08 ± 1.64% kg, *p* = 0.41), dry lean mass (LEU: 0.29 ± 0.29 kg, DILEU: 0.38 ± 0.32 kg, PLA: 0.26 ± 0.60 kg, *p* = 0.78), total body water (LEU: 0.78 ± 0.68 L, DILEU: 0.96 ± 0.83 L, PLA: 0.92 ± 1.29 L, *p* = 0.90), intracellular water (LEU: 0.54 ± 0.47 L, DILEU: 0.67 ± 0.52 L, PLA: 0.63 ± 0.65 L, *p* = 0.89), and extracellular water (LEU: 0.25 ± 0.27 L, DILEU: 0.29 ± 0.34 L, PLA: 0.29 ± 0.35 L, *p* = 0.93).

**Conclusion**: Supplementation with dileucine or leucine for 10 weeks while completing a resistance training program resulted in similar changes in body mass, body composition, and body waters as what were observed in a placebo.

**Acknowledgments**: This study was funded by an unrestricted grant from Ingenious Ingredients, LP (Lewisville, TX USA).

**Conflicts of Interest**: Ralf Jäger, PhD and Martin Purpura, PhD have filed and received patents regarding dileucine supplementation and are founders of Ingenious Ingredients LP, the sponsor of this study. Neither individual played any role in collecting, analyzing, or interpreting the data from this study protocol.

**Effects of 10 Weeks of Dileucine Supplementation on Athletic Performance**

Anthony M. Hagele^a^, Joesi M. Krieger^a^, Joshua Iannotti^a^, Kevin F. Holley^a^, Paige Sutton^a^, Leah Allen^a^, Connor J. Gaige^a^, Logan S. Orr^a^, Ralf Jäger^b,c^, Martin Purpura^b,c^,Chad M. Kerksick^a^*

^a^Exercise and Performance Nutrition Laboratory, Department of Kinesiology, Lindenwood University, St. Charles, MO, USA; ^b^Increnovo, Whitefish Bay, WI USA; ^c^Ingeniuos Ingredients L.P., Lewisville, TX USA

*Corresponding author: ckerksick@lindenwood.edu

**Background**: Absorption of amino acids, particularly the essential amino acids, is known to be a critical factor that influences changes in muscle protein synthesis rates leading to augmentation of adaptations to resistance training. Significant research has recommended an increased intake of dietary protein along with adequate intake of the essential amino acid, leucine. Other research has indicated that ingestion of dileucine, a leucine–leucine dipeptide, can favorably increase circulating leucine levels and support maximal rats of muscle protein synthesis. Research, however, has yet to examine if dileucine supplementation can augment resistance training adaptations resulting in an increase in performance. The purpose of this study was to examine the impact of 10 weeks of dileucine supplementation has on changes in upper and lower-body maximal strength and endurance in resistance trained males.

**Methods**: 32 resistance trained males (28.4 ± 6.0 years, 176.3 ± 7.4 cm, 79.7 ± 12.5 kg, 25.7 ± 3.9 kg/m2, 19.0 ± 5.7% fat) were randomly assigned to ingest in a double-blind fashion either 2 grams of dileucine monohydrate (RAMPS™, Ingenious Ingredients, DILEU), 2 grams of leucine (LEU), or 2 grams of resistant starch as a placebo (PLA) for 10 weeks. Participants were assigned to complete a 4 day per week, split-body resistance training program targeting every major muscle group across 3-4 sets of 6- 10 repetitions at intensities from 70 – 85% 1RM. After 0, 2, 6, and 10 weeks of supplementation, participants were evaluated for changes in one-repetition maximum (1RM) and repetitions to failure (RTF) using the leg press (LP) and bench press (BP) exercises, as well as anaerobic capacity using the Wingate test and maximal voluntary contractions (MVC) using an isometric mid-thigh pull. Data were analyzed using mixed factorial ANOVAs using SPSS and one-way ANOVA using change scores from baseline. Data in presented as means ± SD.

**Results**: Significant main effects for time (p < 0.001) were realized for LP and BP 1RM as well LP and BP RTF providing evidence of a sound resistance training stimulus. A significant group x time interaction was identified (p = 0.027) for the changes in LP 1RM while no significant group x time interaction was observed for BP 1RM (p = 0.165). Tukey post-hoc comparisons revealed that greater increases were observed in DILEU when compared to PLA (95% CI: 5.6, 78.2 kg, p = 0.021), while no changes were observed between the other groups. Changes in LP RTF exhibited a significant group x time interaction (p = 0.047) while no significant interaction was observed for BP RTF (p = 0.11). Tukey post-hoc comparisons revealed that DILEU tended (95% CI: −0.21, 21.1 reps, p = 0.055) to complete greater repetitions when compared to LEU after completion of the study protocol. No significant group x time interaction effects were identified for Wingate peak power (p = 0.09), average power (p = 0.23), fatigue rate (p = 0.78), and total work (p = 0.23). Additionally, no significant group x time interaction was identified for isometric MVC (p = 0.33). A significant time effect was identified for isometric MVC indicated the peak force production increased similarly in all groups as a result of the study protocol.

**Conclusion**: Supplementation with 2 grams of dileucine for 10 weeks in healthy, resistance trained males leads to greater increases in lower body maximal strength when compared to placebo ingestion and tended to result in a greater number of leg press repetitions being performed when compared to leucine ingestion. All changes observed with leucine ingestion were similar to changes observed when a placebo was consumed.

**Acknowledgments**: This study was funded by an unrestricted grant from Ingenious Ingredients, LP (Lewisville, TX USA) .

**Conflicts of Interest**: Ralf Jäger, PhD and Martin Purpura, PhD have filed and received patents regarding dileucine supplementation and are founders of Ingenious Ingredients LP, the sponsor of this study. Neither individual played any role in collecting, analyzing, or interpreting the data from this study protocol.

**Table 1.** Sustained attention/reaction time, hand steadiness, and mood.

	Control	Energy Drink	*p* value
PVT (msec)	297 ± 31	294 ± 28	0.6174
False Starts	2.9 ± 3.0	2.2 ± 1.9	0.1966
Hand Steadiness Errors: Number of Touches	8.5 ± 6.7	8.9 ± 9.7	0.9999
Hand Steadiness Errors: Total Contact Time (seconds)	1.2 ± 1.4	0.9 ± 1.0	0.3836
Total Mood Disturbance Score	19.8 ± 21.3	16.5 ± 18.5	0.3086
Vigor	12.7 ± 5.7	15.2 ± 4.8	0.1307
Fatigue	6.8 ± 4.6	6.2 ± 4.7	0.8564

**Table 1.** Weigh-in vs. fight-weight.

	Official Weigh-In (kg)	Fight-Weight	% Change	p-value
N = 38:	119.1 ± 8.8	117.2 ± 4.7	−1.0 ± 0.5	P = 0.462

Date is expressed as the mean ± SD; *Significance set at P ≤ 0.05

**Table 1.** 24 HOURS PRE VS OFFICAL WEIGH-IN.

	24 HOURS PRE (kg)	OFFICIAL WEIGH-IN (kg)	% CHANGE	P-VALUE
N = 128:	59.3 ± 4.3	56.7 ± 4.0	−4.6 ± 0.7	P < .001

Mean ± SD, *Significance set at P ≤ 0.05

**Table 1.** Time to completion for firefighter-specific tasks and the MAS test.

	Group	Baseline	Post-Testing	*p* value
Hose Line Advance	Control	9.3 ± 1.3	9.3 ± 2.3	T: 0.202
	Creatine	9.6 ± 1.0	10.1 ± 1.7	GxT: 0.916
Rescue (body drag)	Control	12.5 ± 4.0	12.3 ± 4.0	T: 0.046*
	Creatine	14.8 ± 2.5	13.3 ± 2.5	GxT: 0.017*
Stair Climb	Control	30.9 ± 6.7	27.9 ± 5.8	T: 0.058
	Creatine	30.2 ± 4.9	30.3 ± 3.9	GxT: 0.041*
Forcible Entry	Control	15.8 ± 4.4	17.4 ± 5.4	T:0.914
	Creatine	17.8 ± 6.1	17.0 ± 5.8	GxT: 0.017*
Total Completion Time	Control	68.4 ± 12.0	66.9 ± 14.6	T: 0.134
	Creatine	72.3.4 ± 12.0	70.8 ± 11.6	GxT: 0.624
MAS	Control	351.5 ± 34.4	321.2 ± 18.0	T: < 0.001*
	Creatine	333.4 ± 15.2	320.6 ± 14.2	GxT: 0.079

Data are presented as mean ± SD for time to completion (in seconds). *Denotes significance at *p* < 0.05.

